# Exploring the variances of climate change opinions in Germany at a fine-grained local scale

**DOI:** 10.1038/s41467-024-45930-8

**Published:** 2024-02-29

**Authors:** Lars Mewes, Leonie Tuitjer, Peter Dirksmeier

**Affiliations:** 1 Institute of Economic and Cultural Geography, Leibniz University Hannover, Hannover, Germany; 2 Artec—Research Center for Sustainability, Bremen University, Bremen, Germany

**Keywords:** Geography, Climate change

## Abstract

How and why climate change opinions vary within countries at a small geographic scale is rarely investigated. Previous research has focused on public opinions at the individual or national level, leaving local differences within countries and their underlying factors largely unexplored. The lack of research at subnational levels is problematic, as adaptation and mitigation policies depend on collective support and action involving multiple stakeholders at the local scale. It is thus crucial to identify geographic differences in climate change opinions and to unravel their determinants at a fine-grained local scale. We examine public CCOs across 4,667 municipalities in Germany by relying on a representative survey of households. Here we show substantial and systematic differences in public climate change opinions across locations that manifest between urban vs. rural and prospering vs. declining areas. Besides these geographic features, more complex historical and cultural differences between places play an important role.

## Abstract (web summary)

Mewes and colleagues show substantial and systematic differences in public climate change opinions across Germany that manifest between urban vs. rural and prospering vs. declining areas. Besides these geographic features, more complex historical and cultural differences between places play an important role.

## Introduction

A small number of previous studies has shown that public climate change opinions (CCOs) can vary substantially at the local scale^1–3^. The local variation in aggregate opinions within the same country is important for at least two reasons. First, public awareness of climate change has grown around the world^4,5^, but many countries experience stagnating opinions paired with trends of growing skepticism^4–6^. People continue to disagree on climate change resulting in strong polarization. The empirical evidence on geographically varying CCOs within a country indicates that this polarization manifests at the local level, causing strong interregional divergence between places leading and lagging in CCOs^7,8^. Second, geographic polarization is problematic as it increases inequalities and creates tensions between places. Geographic polarization also impedes the efficient implementation of climate change policies that depend on collective support and action at small spatial scales involving multiple stakeholders such as households, businesses, or governments^5,9–17^. CCOs do not translate into climate-friendly lifestyles automatically, but awareness represents an important prerequisite for climate-related actions^18^. Knowing where and understanding why people have different CCOs in different places thus greatly enhances our understanding of the mechanisms that contribute to or inhibit the achievement of climate goals at local levels.

Yet, the geographic polarization of CCOs at the local scale remains rarely investigated. Reasons for the observed local variation in CCOs remain unexplored and the empirical evidence is still restricted to a single country. Previous research has concentrated on revealing geographic variation in CCOs across locations in the United States (US)^1–3^. These findings have greatly advanced our understanding of the geographic dispersion of public CCOs within a country. However, they may be specific to the US. The US has important peculiarities, including its size (the US ranks fourth regarding land area and third regarding population), its geographical diversity (coastal vs. inland, hot vs. cold, low-lying vs. mountainous areas) as well as socioeconomic diversity (ethnically heterogeneous vs. homogeneous, growing vs. declining, urban vs. rural areas), and its political landscape (Democrat vs. Republican areas). Specifically, the two-party system petrifies ideological boundaries between people and increases partisanship in public opinions, including climate change that manifest at a local scale^1,5,19^. These features specific to the US increase the likelihood of identifying varying CCOs within one country and are thus difficult to generalize across a geographically, politically, economically, and culturally diverse planet.

Besides the lack of empirical evidence on other geographic contexts, we know little about the underlying factors that are responsible for the geographic dispersion of public CCOs across a country. Previous research^1,2^ has focused on explaining local differences by projecting results from individual-level surveys to the local level. Individual-level examination has shown that sociodemographic features such as gender, age, education, income, or political beliefs predict individual CCOs^4,20,21^. As people with similar sociodemographic characteristics tend to cluster geographically^8^, the observed geographic differences in CCOs are likely the outcome of varying sociodemographic compositions of places. Since the expected relationship between individual-level predictors is assumed to be constant across places (i.e., individual income predicts people’s CCOs in place A to the same extent as in place B, holding additional sociodemographic predictors constant), the sociodemographic variation between places, however, is insufficient to explain differences in CCOs between places of similar sociodemographic structure. Besides social and demographic factors, a systematic identification of contextual dynamics that are associated with the geographic variation in CCOs is necessary to gain a better understanding of how local variation in public CCOs emerges.

We complement previous research that has focused on identifying geographic differences in CCOs by investigating the contextual factors that may be associated with them. We test four contextual factors specifically. The first geographic factor concerns a possible urban-rural divide in CCOs. Classic work suggests that urban living due to size, diversity, and density of cities produces attitudes distinct from those in rural areas^22^. This context effect implies that urban living makes people more aware of climate change. Cities provide better access to a large spectrum of diverse information, knowledge, and attitudes, which spread fast via social networks. The context effect is often tightly coupled with a composition effect, as people who seek access to city amenities move to them. Accordingly, an urban-rural divide in CCOs is likely, because the population in cities is, on average, younger, better educated, and more left-leaning (i.e., socio-demographic factors associated with CCOs)^20^ than in rural areas. The higher concentration of CCOs, in turn, likely influences others in their opinion building via social impact^23^. Previous studies on the urban-rural divide find mixed results for environmental attitudes more generally (with no clear focus on climate change)^24^. The existence of an urban-rural divide concerning CCOs is confirmed for China^6^. Given these theoretical considerations and empirical findings we expect that people in urban areas are more likely aware of climate change than people in rural areas (hypothesis 1).

Second, interregional divergence is widening between prospering and declining areas in many countries around the world^7,25,26^. Geographic polarization due to uneven economic development leads to differing or even opposing public mass opinions between places. In fact, research on the geography of discontent finds that people in declining areas show disproportionately high support for right-wing populism^27,28^. A persistent narrative of populism is to downplay or even deny climate change^29,30^. The hostility and scepticism sowed by populist parties meets the fertile ground in economically deprived areas, where people feel left behind^25^. However, it is largely unknown whether economic polarization between places manifests itself in CCOs. Research on environmental awareness reports ambiguous results regarding the hypothesis that concern for the environment increases with the wealth of a nation^31–34^. Here, we focus on climate change opinions at the subnational level. Specifically, we hypothesize that people in prospering regions are more likely aware of climate change than people in declining areas (hypothesis 2).

Third, places develop more complex and distinct cultures that constitute and structure the social interaction of people in their local contexts^35^. Local cultures, defined as collectively shared values and beliefs^36^, establish place-specific social norms influencing people’s preferences and behaviors. These, in turn, contribute to the continuous socialization of individuals^37^. In so doing, people’s climate change awareness depends, besides their individual predisposition, on their local context via social impact^23,38^. That means if climate change opinion is a valued cultural feature in location A and not in B, individuals in A are more likely to be aware of climate change than people in B, even though they do not share a political green attitude (i.e., a strong individual-level predictor of CCOs^20^). As people transmit these cultural traits through social interactions from generation to generation^39^, local cultures are unlikely to change in the short-run. Hence, higher or lower climate change opinion in some places today is likely associated with the geographic concentration of certain cultures favoring or hindering an increase in awareness. The existence of path-dependent local cultures has been demonstrated empirically for specific individual attitudes, including trust^40^, policy preferences^41^, or anti-Semitism^42^. How contextual factors, including the manifestation of local cultures, are associated with public mass opinions on climate change and how these contextual factors relate to individual CCOs has not yet been investigated systematically across locations within one country. We expect that people are more likely aware of climate change if they live in places where green attitudes represent an established cultural norm (hypothesis 3).

We address these gaps by focusing on contextual factors responsible for the observed variation in public CCOs, while controlling for most important individual-level predictors in a multilevel regression analysis. In addition, we overcome the focus on the US in previous research on public CCOs^1–3^ by providing systematic assessment of geographic differences in public CCOs in Germany, Europe’s largest economy. Germany represents an interesting case, because it has been perceived as a role model within climate change politics^43–45^. Since 1990, Germany has implemented national climate protection and emission reduction targets^46^, increasing, for example, its large-scale investment in renewable energies to meet national as well as international climate goals^47^. Moreover, due to Germany’s influence in European climate politics, the EU’s engagement in international environmental and climate change politics is strongly coupled to Germany’s support and activities for which public opinions are key^48^. This role model perception, however, also conflicts with general public opinions as indicated by the growing vote share of right-wing populist and climate change-denying parties^29,30^. In addition, Germany is substantially different to the US in terms of its land area (Germany takes up half of the land size of Texas), political landscape (multi-party instead of two-party system), and its history (Germany was divided into two politically different countries between 1949 and 1990, which led to different institutional legacies in CCOs).

East and West Germany followed distinct environmental policies and ideologies^49–51^. During the division of Germany, environmental movements emerged in the 1970s and 1980s due to growing environmental awareness internationally (e.g. Club of Rome in 1968) as well as nationally (e.g. antinuclear power movements in West Germany^52,53^). The democratic system in West Germany allowed citizens to engage in environmental activism during these years, whereas environmental organizations were not allowed in East Germany. While such movements gained public popularity and political influence in West Germany, such organized activism was impossible in East Germany^51^. There was some environmental engagement in East Germany, for example, organized in the Cultural Association of the GDR (Kulturbund); however, national leaders were effective in restricting its influence to a minimum^50^. Its scope cannot be compared to the environmental movements in West Germany. The risk of political persecution, which limited the development of organized and collective social-environmental movements in East Germany, might still contribute to the differences in CCOs between both parts of the country. Empirical research has clearly evidenced that in Eastern European countries CCOs^21^, and environmental concerns^49^ are lower than in Western European countries. Thus, we hypothesize that distinct CCOs between East and West Germany still exist today (hypothesis 4).

We collect information on public CCOs from a representative household survey (Green Socio-Ecological Panel (Green SOEP)), conducted by the Leibniz Institute for Economic Research between 2012 and 2015 (including 4 waves)^54–59^. The Green SOEP provides detailed information on German households (*n* = 12,612), including their sociodemographic characteristics, geolocations, and opinions on climate change (see Supplementary Note 1). There is no scientific consensus on how to assess CCOs in the literature^4^. Climate change opinion is multifaceted, including various dimensions such as peoples’ understanding of climate change, their concern about it, or their support for climate change policies. By examining CCOs, researchers are confronted with selecting specific dimensions of climate change opinions from a set of survey questions to assess them. Researchers may select several survey questions to cover one dimension (e.g., climate change skepticism^60^), or they take a broader perspective and use several survey questions that cover multiple dimensions^1,5,61^. In this article, we take a broader perspective on climate change to assess opinions. Specifically, we use three established items^1,4–6,20,21^ reporting a household’s belief in the existence of climate change (*n* = 11,903), their concern for its possible consequences (*n* = 8,487), and their perceived importance of collective responses (*n* = 12,337) (see Methods and Supplementary Note 2). At the national level, our data indicates that 82% of respondents believe that climate change has already begun, 58% are concerned, and 85% perceive collective action as important, but as our geographic analysis demonstrates, local estimates vary considerably.

## Results

To assess accurate geographic differences in public CCOs, we used detailed information on households’ places of residence to the level of the 4,667 municipalities that represent a very fine-grained administrative level in Germany. Uncovering public opinions at a small spatial scale is methodologically challenging. One possibility is to disaggregate the data (i.e., simply calculating regional averages based on households’ locations). Disaggregation suffers from absent or imprecise estimates in low-population areas. Another approach is to use multilevel regression with poststratification (MRP)^1,62^. Besides the variable of interest, MRP requires the joint distribution of sociodemographic predictors at the same geographic scale^63^. In many research contexts, these information is not available at very small spatial scales. We therefore applied a spatial smoothing function with Actor-Based Clustering^64^ as an alternative approach.

In short, this approach uses the smallest scale of spatial information available in the data (here, which of the 4667 municipalities a household resides in) to depict geographic patterns without imposing any predetermined higher-level spatial boundaries. Specifically, the CCO scores for each municipality *i* were calculated based on CCO scores of households residing in municipality i as well as households in all municipalities *j* (*j* ≠ *i*) by considering spatial weights such that geographically proximate households receive higher weights than distant ones. For geographically weighting responses, we calculated the distance between all 4667 × 4667 German municipalities and transformed the geographic distance into spatial weights using a log-logistic distance-decay function following previous research^64–67^ (see Methods for details).

To quantify the accuracy of actor-based clustering and the applied spatial smoothing function, we followed previous work^1,62^ and cross-validated public CCOs as estimated with actor-based clustering by simulating small sample sizes based on subsets from large sample sizes in more populated regions. Cross-validation demonstrated that actor-based clustering produces highly accurate measures with mean absolute errors ranging between 0.38–1.35% (belief), 0.57–3.79% (concern), and 2.25–4.27% (importance) (see Supplementary Note 3 and Supplementary Figs. 1 and 2). The range of errors is comparable to methods applied in earlier research, such as MRP^1^. We excluded 96 municipalities for which the data was too sparse (<1% of total population in Germany) from the geographic mapping, reducing the number of municipalities to 4,571 (see Supplementary Note 4 and Supplementary Figs. 1 and 2).

**Fig. 1:**
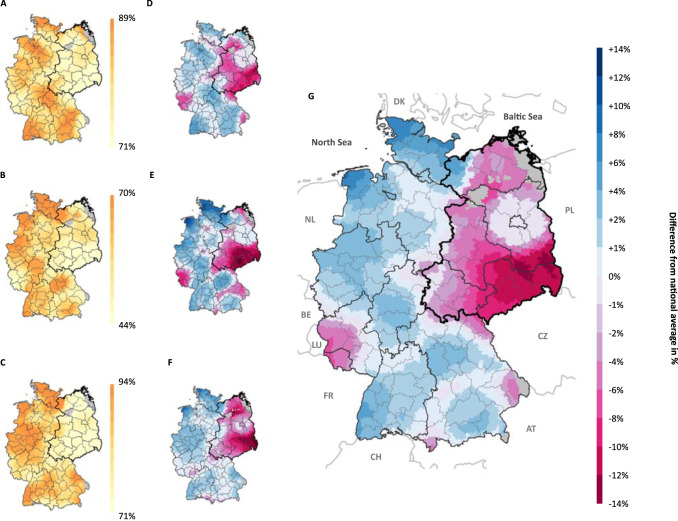
Geographic distribution of public CCOs across German municipalities. The shares of local populations that (**A**) believe climate change has already begun, (**B**) are concerned, and (**C**) perceive collective responses to be important. The percentage difference from the national average is visualized in panel (**D**) for awareness, (**E**) concern, (**F**) importance. The average difference from the national average across the three dimensions reported in (**D**–**F**) is depicted in (**G**). The bold black line indicates the former division into East and West Germany. Solid black lines indicate the 16 federal states. Solid grey lines indicate the 96 planning regions used for the multilevel estimations. Municipalities with too little information are colored in grey (see Supplementary Note 5).

We applied the spatial smoothing function to estimate public CCOs across the final sample of German municipalities (*n* = 4571) as depicted in Fig. 1. The figure shows the share of the local population (and the difference from the national average in percent) who believe that climate change has already begun (panels A and D), who are concerned about its consequences (panels B and E), and who perceive collective action against it as important (panels C and F). All maps show substantial and relatively stable geographic clustering across all CCO dimensions within Germany with regional shares varying between 71–89% (belief), 44–70% (concern) and 71–94% (importance). These observed magnitudes in public CCOs are comparable to the local variation reported in the US^1^. The combination of all three CCO dimensions (panel G), that is the average of panels D-F for every municipality, accounts for 83% of the observed geographic variance in public CCOs as indicated by principal component analysis, substantiating the robustness of the results across the dimensions.

We validated our findings obtained from spatial smoothing by applying MRP at the level of 96 German planning regions (see Supplementary Note 5). Planning regions (*“Raumordnungsregionen”*) are functional units capturing socioeconomic interactions (e.g., core-periphery commuting) that cross administrative boundaries. The correlation r = 0.7, CI = [0.58; 0.79], and *p* < 0.001 of regional estimates obtained from MRP and spatial smoothing indicates that both methods produce acceptably similar results. We also validated our findings concerning the four geographic features using MRP. The revealed geographic patterns of public CCOs are therefore not an artefact of the spatial smoothing function but validated by using MRP as a fundamentally different methodology.

### Differences between urban and rural areas

**Fig. 2:**
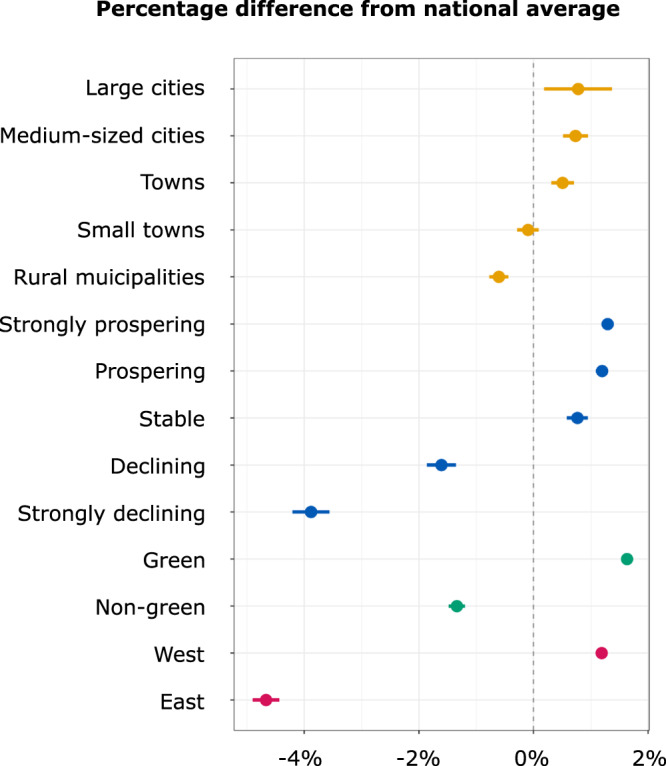
Grouped differences from national average in public CCOs. Local shares of people who believe climate change has already begun, are concerned, and perceive collective responses to be important were calculated at the municipality level as percentage difference from the national mean. Error bars represent 95% confidence intervals of SEM.

To first investigate whether differences between urban and rural areas exist, we used a predefined categorization of municipalities into five settlement types as provided by the Federal Institute for Research on Building, Urban Affairs, and Spatial Development (BBSR):^68^ (1) large cities *(n* = 79, 1.8% of total population), (2) medium-sized cities (*n* = 621, 14.1%), (3) towns (*n* = 868, 19.7%), (4) small towns (*n* = 1211, 27.5%), and (5) rural municipalities (*n* = 1624, 36.9%). This categorization is based on municipalities’ population sizes, their functions, and settlement structures. 168 municipalities (0.01% of total population) were not classified. We calculated the percentage difference from the national average and corresponding 95% confidence intervals for each settlement type across the individual CCO dimensions. Results are depicted in Fig. 2 and indicate significant differences between more urban areas and more rural ones. The overall difference in public CCOs (panel D) between large cities (type 1) and rural municipalities (type 5) is ~2%. The results of one-way analysis of variance (ANOVA) indicate that group differences across the five settlement types are significant for all CCO dimensions (belief: F = 10.4, *p* < 0.001; concern: F = 33.4, *p* < 0.001; importance: F = 26.6, *p* < 0.001; overall: F = 28.8, *p* < 0.001).

### Differences between prospering vs. declining areas

Our second hypothesis concerns geographic dispersion of public CCOs between prospering vs. declining areas. We again relied on a classification provided by the *BBSR*^68^ that groups municipalities into five categories: (1) strongly prospering (*n* = 867, 19.8% of total population), (2) prospering (*n* = 1484, 33.9%), (3) stable (*n* = 605, 13.8%), (4) declining (*n* = 876, 20%), and (5) strongly declining (*n* = 542, 12.4%). This categorization is derived from six indicators (population development, net migration, development of workforce aged 20–64, workplace development, unemployment rate, development of commercial tax income) and therefore provides a comprehensive distinction between prospering and declining areas. 197 municipalities (0.01% of the total population) were not classified. The results, depicted in Fig. 2, indicate a significant difference between prospering and declining municipalities in public CCOs. While the prospering municipalities all show positive differences from the national average, the percentage difference is negative for declining areas. The overall differences between the most prospering and most declining regions is 6.4% and hence exceeds the observed urban-rural divide by a factor of 3. As indicated by a one-way ANOVA test, the group differences are significant for every CCO dimension (belief: F = 405.8, *p* < 0.001; concern: F = 371.7, *p* < 0.001; importance: F = 326.1, *p* < 0.001; overall: F = 477.1, *p* < 0.001).

### Local green cultures

**Fig. 3:**
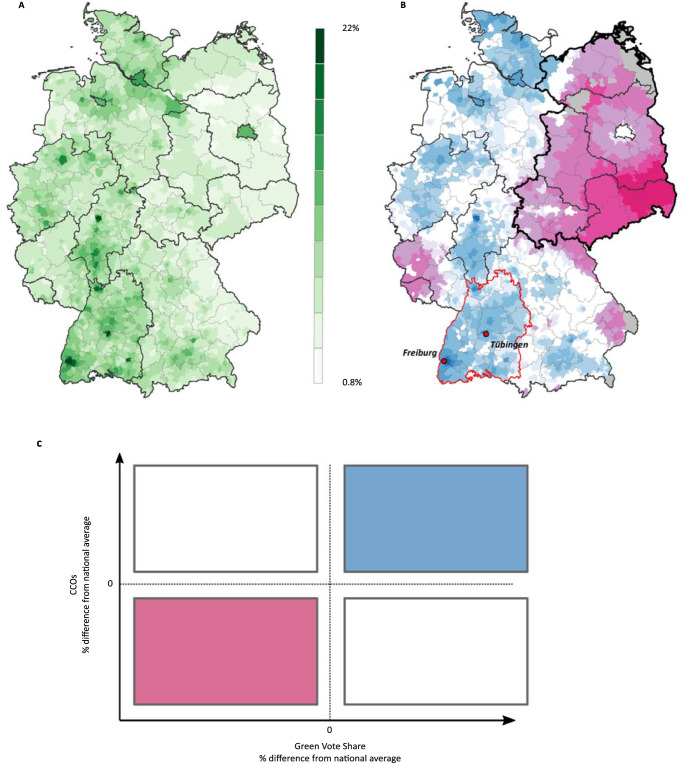
Local green cultures and their spatial relationship with public CCOs. Local green cultures and their spatial relationship with public CCOs as depicted by local green vote shares in 1994 (**A**), the local pairing of above/below average green vote shares and above/below average CCO shares (**B**). The color scheme of panel **B** is illustrated in panel **C**.

We examined the relationship between pre-existing differences in local green political cultures and current CCOs to address hypothesis three. To approximate green political cultures, we relied on local variation in votes for the Green Party during the general election in 1994. The 1994 federal election was the first election after the Greens from the West merged with the civil rights party Alliance 90 from the East and ran as an all-German party (Alliance 90/The Greens) with a clear focus on environmental topics^53^. Although climate change became an established topic for the German public during the late 1980s and early 1990s^43^, the Green Party’s focus on global warming and the fight against climate change did not play a meaningful role in national elections before 2002^69,70^. Hence, local variation in green votes in 1994 indicates to what extent local populations favored green policies as an expression of general environmental concern roughly twenty years before the household survey was conducted. Figure 3 displays the local share of green votes in 1994 (panel A), with a national average of 5.8% and local shares ranging between 0.8% and 22%. To test group differences, we divided the sample into “green” (above-average vote shares) and “non-green” municipalities (below-average vote shares). As depicted in Fig. 2, green municipalities have distinct CCOs than nongreen municipalities. The results of two sample t-tests indicate that group differences are significant for all CCO dimensions (belief: t = 31.8, *p* < 0.001; concern: t = 37.1, *p* < 0.001; importance: t = 27.2, *p* < 0.001; overall: t = 37.3, *p* < 0.001).

To provide a visual illustration of the relationship between local green vote shares and overall CCOs (panel G of Fig. 1), we created a map (panel B of Fig. 3) highlighting the local co-occurrence of either positive or negative differences from the national average in both variables (70% of all municipalities). In this map, we highlighted the cities Freiburg and Tübingen in Germany’s Federal State Baden-Wuerttemberg (red border in Fig. 3), which show above-average values in local green vote shares and local CCOs. In these cities, local policymakers have long-established green development strategies^71^. Baden-Wuerttemberg is the first federal state in German history to have a Minister-President from the Green Party. The local variation in public CCOs today seems to be associated with the manifestation of local green political cultures in the past. More generally, the correlation between local shares of green votes and CCOs are constant across all CCO dimensions (belief: r = 0.5, *p* < 0.001, concern: r = 0.47, *p* < 0.001, importance: r = 0.4, *p* < 0.001). Between 16% and 25% of the observed local variation in public CCOs at the municipality level is explained by the presence of local green political cultures.

### Differences between East and West Germany

In our fourth hypothesis, we expect distinct CCOs between East and West Germany. Over 20 years after German reunification, the geographic variation in public CCOs (see maps in Fig. 1) shows a striking East-West divide. The polarization between East and West is prevalent in each CCO dimension, although to varying degrees. Municipalities in West Germany show decisively higher levels of belief (West: 82.9%, CI = [82.9–83.0%] vs. East: 78.0%, CI = [77.8–78.3%]), concern (West: 58.3%, CI = [58.2–58.4%] vs. East: 51.2%, CI = [50.9–51.5%]), and perceived importance of collective responses (West: 85.3%, CI = [85.3–85.4%] vs. East: 79.8%, CI = [79.5–80.0%]). Group differences are significant as indicated by the results of two sample t-tests (belief: t = 41.6, *p* < 0.001; concern: t = −41.5, *p* < 0.001; importance: t = −43.0, *p* < 0.001; overall: t = −47.7, *p* < 0.001).

**Fig. 4:**
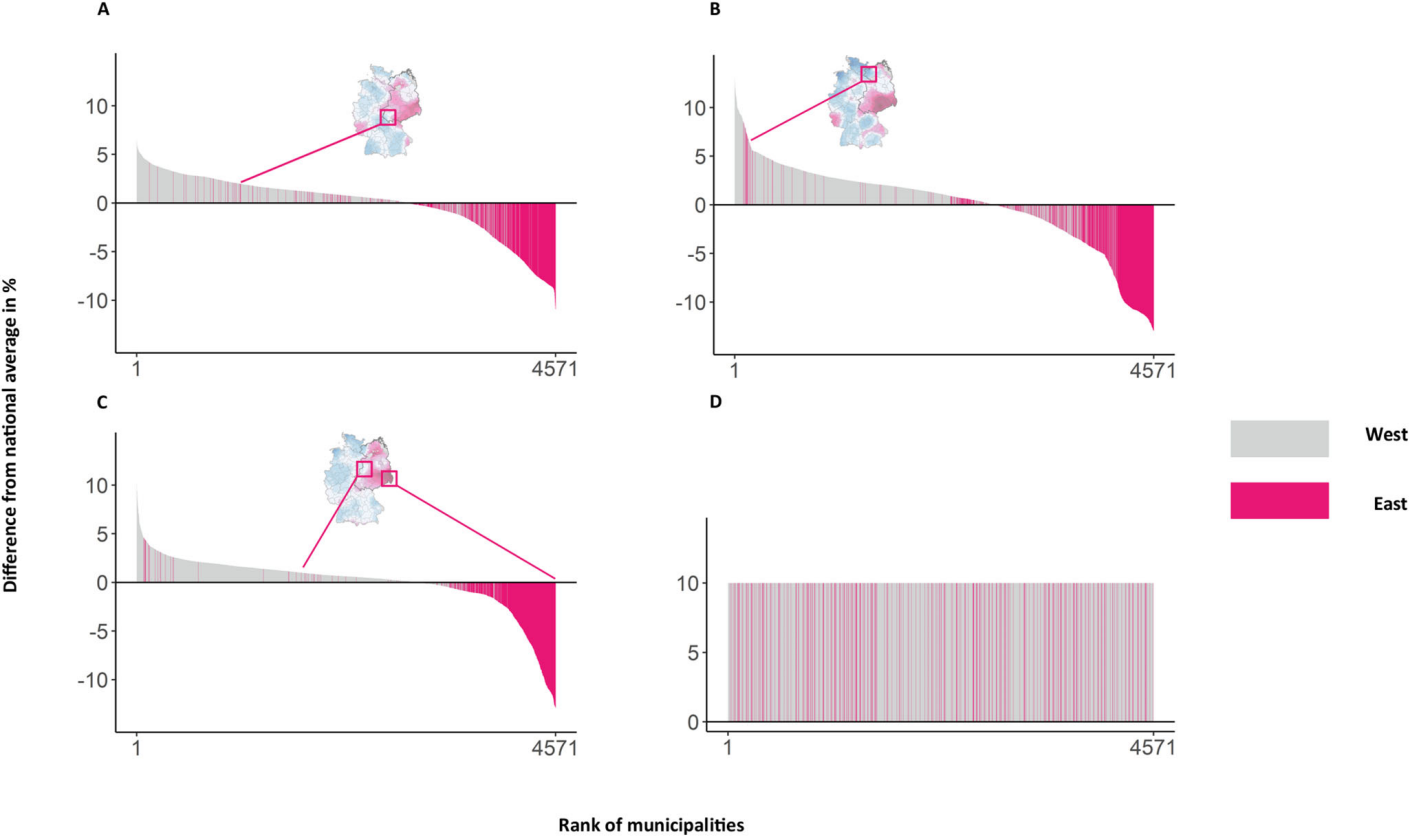
Ranked deviations from the national average in public CCOs across municipalities in East and West Germany. Ranked deviations from the national average in public CCOs across municipalities in East and West Germany for (**A**) belief, (**B**) concern, and (**C**) importance. To ease interpretation, we added a random ordering of municipalities (**D**).

The systematic polarization of public CCOs between municipalities in East (*N* = 927, 20.3% of all municipalities) and West (N = 3,644, 79.7%) becomes even more evident by investigating the distribution of local CCOs in more detail. In Fig. 4, municipalities are ranked according to their deviation from the national average across the three CCO dimensions of belief (panel A), concern (panel B), and importance (panel C). We added a random ordering of municipalities (panel D) to highlight the systematic difference in CCOs between East and West. Of the 2,950 municipalities with an above-average belief in climate change (positive difference from the national average as shown in panel A of Fig. 4), 116 are located in the East representing 12.5% of all Eastern municipalities. Regarding concern (panel B) and importance (panel C), the share of Eastern regions with above-average shares is 17% and 5%, respectively. Specifically, in 95% of the municipalities located in East Germany, local populations place less importance on collective responses to climate change than the national average. Dividing the country once again into East and West explains 53% of the observed variance in public CCOs. Hence, public polarization in climate opinions manifests to a large degree between East and West Germany.

As outlined above, we investigated how contextual factors (urban vs. rural, prospering vs. declining, green vs. non-green areas, East vs. West) relate to aggregate opinion at the local scale. Individual-level research, however, has shown that individuals’ sociodemographic features, such as their age or education, predict their CCOs^20^. To finally test our four hypotheses, we thus estimated multilevel regression models with households nested in their locations to consider individual-level alongside context-level factors. As the level of municipalities is too fine-grained to estimate multilevel regressions, we nested households into German planning regions (*N* = 96). This regional scale guarantees sufficiently large sample sizes per region (5 < *n* < 431) (see Methods for details). To model households’ opinions across the three CCO dimensions, we created a variable equaling 1 if a household believes in climate change, its members are concerned, and they perceive collective responses as important, and 0 otherwise. Regression results are robust for each CCO dimension separately and (also for) using the original ordinal response scales (see Supplementary Note 6 for additional regression results). Based on previous research^4,20,21^, we included a rich set of household-level variables (gender, age, education, income, political affiliation, environmental concern) representing the most important predictors for individual opinions on climate change. At the regional level, we created four variables each representing one of the four contextual factors (urban vs. rural, prospering vs. declining, East vs. West, green vs. non-green) outlined above (see Methods for a detailed theoretical reasoning of all variables, their definition, and data sources).

Table 1 reports the results. The goodness of fit (R^2^) ranges between .058 (marginal) and .066 (conditional). Consistent with previous individual-level research^4,20,21^, the individual-level predictors are associated with individual CCOs as expected. Females (exp(*β*) = 1.22, *p* < 0.001), people with higher education (exp(*β*) = 1.13, *p* = 0.03), those affiliated to the Green Party (exp(*β*) = 3.58, *p* < 0.001) and environmentally concerned (exp(*β*) = 1.89, *p* < 0.001) show higher odds of being aware of climate change. Individual income (exp(*β*) = 1.98, *p* < 0.001) did not play a meaningful role in our estimations. Political affiliation (~258% higher odds of being aware of climate change) and environmental concern (~89% higher odds) show the strongest relationship with climate change awareness validating previous research^20^.

**Table 1:** Regression results of generalized linear mixed models

	Without local context	Urban vs. Rural	Prospering vs. Declining	Green vs. Non-green	Green individual vs. Green regional	East vs. West
	exp(*β*) (P) [95% CI]	exp(*β*) (P) [95% CI]	exp(*β*) (P) [95% CI]	exp(*β*) (P) [95% CI]	exp(*β*) (P) [95% CI]	exp(*β*) (P) [95% CI]
*Household level*						
*(Intercept)*	1.09 (0.211)	1.07 (0.337)	1.09 (0.227)	1.06 (0.358)	1.06 (0.363)	1.21 (0.007)
	[0.95; 1.25]	[0.93; 1.23]	[0.95; 1.24]	[0.93; 1.22]	[0.93; 1.22]	[1.05; 1.39]
*Sex*	0.82 (<0.001)	0.83 (<0.001)	0.83 (<0.001)	0.83 (<0.001)	0.83 (<0.001)	0.81 (<0.001)
*(1=Male)*	[0.74; 0.91]	[0.75; 0.92]	[0.75; 0.91]	[0.75; 0.92]	[0.75; 0.92]	[0.74; 0.90]
*Age*	1.58 (0.007)	1.58 (0.007)	1.63 (0.004)	1.63 (0.004)	1.63 (0.004)	1.58 (0.007)
	[1.13; 2.20]	[1.14; 2.21]	[1.17; 2.27]	[1.17; 2.28]	[1.17; 2.28]	[1.14; 2.21]
*Age (Squared)*	0.62 (0.005)	0.62 (0.005)	0.60 (0.003)	0.60 (0.003)	0.60 (0.003)	0.62 (0.005)
	[0.45; 0.87]	[0.45; 0.86]	[0.43; 0.84]	[0.43; 0.84]	[0.43; 0.84]	[0.45; 0.86]
*Education*	1.13 (0.030)	1.12 (0.041)	1.14 (0.025)	1.13 (0.033)	1.13 (0.034)	1.16 (0.010)
*(1= High School)*	[1.01; 1.27]	[1.00; 1.26]	[1.02; 1.27]	[1.01; 1.26]	[1.01; 1.26]	[1.04; 1.30]
*Income*	0.99 (0.377)	0.99 (0.418)	0.99 (0.278)	0.99 (0.321)	0.99 (0.321)	0.99 (0.179)
*(1-to-10 scale)*	[0.97; 1.01]	[0.97; 1.01]	[0.97; 1.01]	[0.97; 1.01]	[0.97; 1.01]	[0.97; 1.01]
*Political Affiliation*	3.58 (<0.001)	3.57 (<0.001)	3.54 (<0.001)	3.52 (<0.001)	3.63 (<0.001)	3.52 (<0.001)
*(1=Green Party)*	[2.84; 4.52]	[2.83; 4.51]	[2.80; 4.46]	[2.79; 4.44]	[2.76; 4.79]	[2.79; 4.45]
*Environmental Concern*	1.89 (<0.001)	1.89 (<0.001)	1.87 (<0.001)	1.88 (<0.001)	1.87 (<0.001)	1.86 (<0.001)
*(1* = *ENGO membership)*	[1.63; 2.19]	[1.63; 2.19]	[1.61; 2.17]	[1.62; 2.17]	[1.62; 2.17]	[1.60; 2.15]
***Regional level***						
*Urban*		1.05 (0.068)				
		[1.00; 1.11]				
*Prospering*			1.13 (<0.001)			
			[1.06; 1.19]			
*Green*				1.12 (<0.001)	1.12 (<0.001)	
				[1.06; 1.18]	[1.06; 1.18]	
*East*						0.71 (<0.001)
						[0.62; 0.81]
***Cross-level interaction***						
*Political Affiliation x*					0.95 (0.674)	
*Green*					[0.75; 1.21]	
*AIC*	9893.74	9892.50	9880.83	9881.28	9883.10	9873.19
*BIC*	9955.88	9961.54	9949.87	9950.32	9959.04	9942.23
*R² (conditional)*	0.066	0.066	0.066	0.066	0.066	0.066
*R² (marginal)*	0.058	0.059	0.062	0.062	0.062	0.064
*n individuals*	7361	7361	7361	7361	7361	7361
*n regions*	96	96	96	96	96	96
*Variance (regions)*	0.03	0.02	0.01	0.01	0.01	0.01

Compared with the strongest individual-level predictors, the coefficients of the regional-level predictors range at lower levels but are nevertheless meaningful. The coefficient of urban areas is positive (exp(*β*) = 1.05) but not statistically significant at the 5% level (*p* = 0.068). Given the 95% CI of 1.00 to 1.11, it would be hasty to conclude that an urban-rural divide does not exist^72^. However, the other context factors seem more important as indicated by the regression results. The divide between prospering and declining areas is statistically significant (exp(*β*) = 1.13, *p* < 0.001) and meaningful. If respondents live in more prosperous regional contexts, their odds of being aware of climate change increases by ~13%.

To test hypothesis 3, we included the regional green vote share in 1994 to approximate the existence of local green cultures (see Supplementary Note 7 for similar results for six subsequent general elections). The corresponding coefficient (exp(*β*) = 1.12, *p* <0 .001) indicates that we expect to see a ~12% increase in respondents’ odds of being aware of climate change, for a one-unit increase in the regional green vote share. In theories on local cultures, it is further hypothesized that established values and norms (e.g. environmental concern) are associated with people’s beliefs and behaviors^35,38^. These works raise the important question whether people are more likely aware of climate change if they live in local contexts emphasizing “green” values although they do not hold a green attitude personally. This question has not been systematically investigated regarding public CCOs at a local scale. We investigated the hypothesis by including cross-level interaction terms between the individual-level feature that households tend to vote green and the regional-level predictor of green vote shares. The corresponding results show that local green cultures are particularly associated with people’s opinions about climate change if they personally do not hold a green attitude. That is, the CCOs of a large part of the population (those that do not hold a green attitude a priori) depend on their local context.

Lastly, the divide between East and West remains striking in the multilevel setting. Respondents in East Germany (exp(*β*) = 0.71, *p* < 0.001) have ~30% lower odds of being aware of climate change than Western respondents. Using the individual-level estimate of respondents’ sex as a benchmark (~22 higher odds of being aware of climate change for females) indicates that especially the East-West divide is a meaningful regional-level predictor for individual CCOs. The results for the prospering vs. declining and green vs. non-green areas remained robust even when controlling for the prevalent East-West divide that confounds many socioeconomic variables, including economic development for example (see Supplementary Note 8 for additional model specifications)^73^.

We estimated binary logistic multilevel models with households nested in German planning regions. The dependent variable (CCO) equals 1 if household believes in climate change, is concerned, and perceives collective responses as important; and 0 otherwise. 53% were coded as 1 and 47% as 0. Coefficients represent odd ratios. 95% confidence intervals in square brackets. See Methods for a detailed description of all variables included in the model. All continuous variables were z-standardized beforehand. See Supplement for additional regression results for each CCO dimension.

## Discussion

This study focused on uncovering geographic polarization in public CCOs at a fine-grained local scale and understanding the underlying factors. We thus extended the existing empirical evidence, previously restricted to the US^1–3^, to a different national setting by analyzing the geographic dispersion of public CCOs across municipalities in Germany. Our results reveal substantial and systematic geographic variation in public CCOs, demonstrating that geographic polarization of public CCOs is not a US-specific phenomenon but also present in a substantially smaller country with a different socioeconomic, historical, cultural, and political context. Second, we enhanced an understanding of how these spatial patterns emerge. Hence, this study systematically assesses the underlying local factors associated with the observed geographic variation in public CCOs. Specifically, we tested four hypotheses while controlling for the most important sociodemographic predictors at the individual level.

Urban and economically prospering areas show substantial higher CCOs than rural and declining regions in Germany. Our results therefore confirm our hypotheses 1 and 2 and validates previous research that hypothesized a potential divide between these types of areas regarding CCOs^1^. The geographic divide points at a more general trend of an increased polarization between places regarding a variety of political and socioeconomic phenomena^7^. The identification of general geographic features that are associated with individual CCOs, such as urbanity or prosperity is helpful to better understand the geography of public CCOs at small spatial scales systematically. By knowing that people show different climate change opinions dependent on their regional context, we are able to better predict CCOs in smaller areas and to limit the list of possible sources that may cause geographic polarization. The identified relationships in this study are correlative and do not indicate causal mechanisms. With that said, we observe people in prospering places are more likely aware of climate change than people in declining places, but we know little about the mechanisms through which public CCOs emerge. Hence, we need to know more about why people in rural or declining places show lower CCOs. Only by studying these questions, the development of appropriate instruments to reduce geographic polarization will be possible.

Besides the distinction of urban vs. rural and prospering vs. declining, our analysis has demonstrated that complex histories and cultures of places are important to understand the geographic dispersion of CCOs. Remarkably, political histories play an important role. By using data on historical election results, we confirm our third hypothesis. Our findings indicate that individual CCOs are higher in regions where people have established strong local green political cultures over time. Even more importantly, people are more likely to be aware of climate change if they live in local contexts where political green cultures have long been established, even though personally they do not prefer to vote green. This result (a) confirms theories on the importance of place-specific cultures on individual beliefs and behaviors^35,38^ and (b) suggests that the local context is especially important for those individuals not already characterized by a pronounced green ideology, which likely is the majority of the population within a country. Based on our survey, 93% of the population (the percentage without a clear green ideology), is therefore likely to be influenced in their opinion by the established green culture in their local context.

Although the data were collected more than 20 years after German reunification, our findings show that it would be hasty to assume that the legacy of socialist environmental politics has ended^49–51^. Obviously, the difference between public CCOs in East and West Germany suggests an effect of the division of Germany on public CCOs that survived at least until 2015—nearly 25 years after reunification. Our results are linked to a growing literature demonstrating an enduring effect of state socialism on individual attitudes and preferences^41,73^. It is not possible with our data to identify the explicit causal effect of the division of Germany on public CCOs, as we only observed households in their current place of residence and not their members’ birth places and distinct biographies. In the early years after reunification, the East Germany population declined rapidly in size, as primarily younger and more educated citizens moved from East to West in search of better job opportunities^74^. Hence, selective migration^75^ might contribute to the strong East-West divide in public CCOs, as research has shown that age and education^20^ play an important role in explaining individual differences in CCOs. To approximate the influence of selective migration on the observed geographic dispersion of public CCOs in Germany, we collected historical data on internal migration, calculated the total net migration for each region early after reunification, and included this variable into our regression analysis. If selective migration had an effect on today’s CCOs, people residing in East German regions that experienced rapid population decline shortly after reunification, would be less aware of climate change than persons residing in East German regions that were less affected by out-migration. Given our robustness check, we did not find evidence of selective migration playing a meaningful role for today’s geography of public CCOs (see Supplementary Note 9) and thus it is likely that the German division has had an effect on individuals’ CCOs. The unique history of Germany allowed us to demonstrate that the socialist legacy regarding environmental politics scales down to the local level and is still present today. This results confirms our fourth hypothesis.

Hence, local cultures are meaningful in explaining local differences in public CCOs. We are clearly demonstrating the need for more rigorous research on local CCOs within various national contexts to avoid blanket assumptions about the spatial distribution of people’s opinion of climate change within one country. The heterogeneity of locations across a country and the importance of the local scale to implement climate change policies demonstrate the need for a more detailed evaluation of public opinions at a local scale. Methodologically, spatial smoothing and multilevel regression with post-stratification (MRP) demonstrate how to use national surveys containing detailed geographic information and public opinions on climate change paired with spatial statistics to provide accurate estimates and to map CCOs across a country’s most fine-grained geographic level.

One important challenge for future research is to investigate changes in local CCOs. In recent years, extreme weather events related to climate change, such as severe heat waves (2018/19) and floods (specifically the 2021 floods in the Ahr Valley in Rhineland-Palatinate, Germany) occurred in Europe. The data in this report was collected between 2012 and 2015. Hence, changes in public opinion caused by these recent weather extremes are not included in the database of this article. However, if and to what extent these weather events caused significant shifts in public CCOs is currently unknown. In fact, empirical evidence of the link between extreme weather events and CCOs is mixed^76^. The growing empirical evidence suggests that extreme weather events have not caused people previously unconcerned about climate change to become concerned, for example. Specifically, a recent study reports no causal effect of extreme weather events on individual climate change awareness for Germany^77^. The mixed evidence may serve as one explanation for the stability of public CCOs. Empirical studies investigating long-term changes in public CCOs indicate that CCOs are relatively stable over time in most Western countries^4,5^. Empirical evidence regarding changes in CCOs at the fine-grained local scale is not available, which represents a pressing research gap. If this research gap can be addressed in future research critically depends on the availability of long-term data providing information on climate change opinions and geolocations of respondents.

The local variation in public CCOs and the importance of place-specific context factors have far-reaching implications for climate change mitigation and adaptation policies, as the local level is crucial for the success of global and national climate action targets^5,9–17^. First, previous research on the local implementation of climate change policies has indicated that places differ in developing action plans^78^ or their implementation^16^. Understanding why places differ regarding their strategies and implementation is important to guarantee the success of global and national targets. Reasons for the observed differences between places, however, remains largely unexplored. The variation in climate change opinions across locations might represent one important factor that is associated with the success of climate change policies at local levels. Second, local variation in public CCOs, suggests an urgent need for climate change policies and their communication to be tailored more specifically to meet local CCOs and local context factors. Having local information on climate change opinions means approaches can be better targeted and avoid “preaching to the choir” and the potential waste of precious public campaign resources in areas already characterized by high CCOs. Third, the need to consider place-specificities when implementing climate change policies at local levels is even more important considering our finding that local individual CCOs depend on the complex history and culture of their local environments. Changing local history is impossible and adjusting local culture seems an inappropriate piece of policy advice, but knowing that these factors are important in understanding public opinions is crucial for policymaking.

## Methods

Data on Climate change opinions (CCOs) was drawn from the Green Socio-Ecological Panel (Green SOEP), a nationally representative survey conducted between 2012 and 2015 by the Leibniz Institute for Economic Research^54–59^. The data set provides information on 12,612 households in total. The respondents were the heads of the surveyed households defined as the person who decides about financial decisions and at least 18 years old. We used three items from the survey to measure opinions on climate change (see Table 2 for a summary and descriptive statistics. Supplementary Notes 1 and 2 provide more details on data collection and survey questions). 7,361 households answered all items and information on their place of residence was given. Households’ places of residence were assigned according to the Official Municipality Key at the level of municipalities that facilitates aggregation to any higher spatial level, such as planning regions or federal states.

**Table 2:** CCO items, their definition, and descriptive statistics

CCO	Survey question	Answers	Recoding	n	Mean
*Belief*	Which of the following statements do you agree with most?	1 = climate change will not occur at all (4.35%), 2 = climate change will take place in the distant future (4.50%), 3 = climate change will take place in the near future (9.02%), 4 = climate change is already taking place (82.12%)	Surveyed households that responded with a 4 were recoded as 1 and all other households as 0	11,903	82.12%
*Concern*	Are you concerned about possible climate change?	1 = not concerned at all (5.64%), 2 (8.20%), 3 (11.17%), 4 (17.30%), 5 (24.52%), 6 (20.02%), 7 = very concerned (13.15%)	Surveyed households that responded with a 5 or higher were recoded as 1 and all other households as 0	8487	57.69%
*Importance*	How important is the fight against climate change?	1 = totally unimportant (1.38%), 2 (3.06%), 3 (10.98%), 4 (30.27%), 5 = very important (54.32%)	Surveyed households that responded with a 4 or higher were recoded as 1 and all other households as 0	12,337	84.59%

Recoding of the belief, concern and importance scales originally included in the surveys. The dichotomous new variables serve as dependent variables in the generalized linear mixed models in Table 1.

### Actor-based clustering with spatial discontinuities

To map public CCOs across German municipalities, we used actor-based clustering with spatial discontinuities^64^. Basically, actor-based clustering allows geographic patterns to emerge from the data by applying a spatial smoothing function:

1 $${{CCO}}_{i}=f({{CCO}}_{k,i},\,{{CCO}}_{k,j},\,{w}_{i,j})$$

The spatial smoothing function estimates local CCOs for municipality *i* based on the opinions of the households *k* (i.e., the actors) residing in *i*. The local CCO for municipality *i* based on households residing in *i* is given by:

2 $${{CCO}}_{i}=\frac{{\sum}_{k\in i}{{CCO}}_{k,i}}{{n}_{i}}$$

with CCO taking 1 if household *k* perceives collective responses as important and 0 if not, for example. ${n}_{i}$ denotes the number of households in municipality *i* included in the sample. Thus, ${{CCO}}_{i}$ represents the share of the population in a given municipality that perceives collective response as important.

**Fig. 5:**
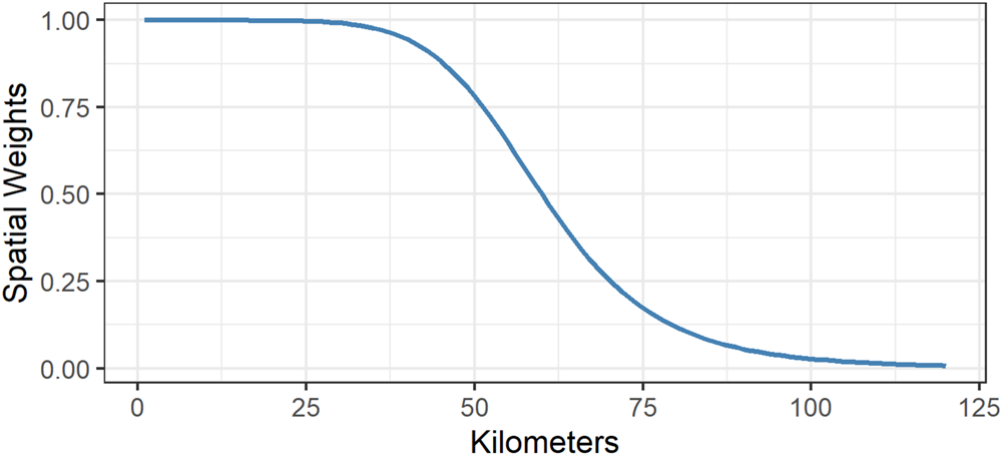
Log-logistic distance-decay function. Log-logistic distance-decay function as used in the spatial smoothing function of actor-based clustering to map CCOs across municipalities with r* =* 60 km and s* =* 7.

As indicated by the spatial smoothing function, local CCOs in municipality *i* are not only based on households residing in *i*, but also on households residing in a different municipality *j* by using spatial weights *w*. For example, households are considered with weight 1 if *j* = *i* and households receive decreasing values <1 if *j* ≠ *i*. There are different ways to calculate spatial weights. Following existing approaches^64–66^, we applied a log-logistic distance-decay function to calculate spatial weights, which takes the following form:

3 $$f\left(d\right)=\frac{1}{1+{\left(\frac{d}{r}\right)}^{s}}$$

where *d* denotes the geographic distance between German municipalities. *r* represents the parameter at which the decay function reaches a value of ½. *s* denotes the slope of the decay function. The applied decay function requires the definition of the unknown parameters *r* and *s*. In our case, *r* depends on the assumed reach of spatial interaction between households residing in distinct municipalities. Existing approaches^64–66^ define spatial interactions based on commuting flows and determine *r* as the maximum distance people are willing to commute between regions as an indicator of spatial interaction. Besides the assumed spatial reach of interactions, *r* is also restricted by the volume of information that flows into the spatial smoothing function. *r* can be set low (e.g. 5 or 10 km) if large-scale data on millions of individuals provides dense information across spatial units in a country. *r* needs to be larger (e.g. >50 km) when sample sizes are smaller to enrich the spatial smoothing function with sufficient information to provide reliable estimates. We defined *r* to be 60 kilometers, which is a reasonable maximum commuting distance in Germany^79^ and provides accurate estimations for less populated municipalities with small or even absent sample sizes (see Supplementary Note 10). Hence, after 60 kilometers, the spatial weight reduces to 0.5. *s* defines the slope of the decay function and therefore determines how fast values decrease with increasing distance after *r* is reached. We defined *s* to be 7. Figure 5 depicts the distance-decay function with *r* = *60* and *s* = *7* used to produce the maps in the main text. Hence, after ~100 kilometers, the spatial weights in our study decrease to a value of nearly 0. Precisely, higher values of *r* and lower values of *s* increase the spatial smoothing while lower values of *r* and higher values of *s* decrease the spatial smoothing. To study the robustness of our results, we varied *r* to take values of 50 and 70 kilometers and *s* to be 6 and 8. The main results are consistent irrespective of *r* and *s* (see Supplementary Note 11).

The final CCO for municipality *i* is thus given by:

4 $${{CCO}}_{i}={{CCO}}_{i}+\frac{{\sum}_{k\in j}{{CCO}}_{{kj}} * {w}_{{ij}} * {n}_{j}}{{\sum}_{k\in j}{w}_{{ij}} * {n}_{j}}$$

where ${n}_{j}$ denotes the number of households in municipality *j* included in the sample. Hence, ${n}_{j}$ weights the final CCOs based on the regional sample sizes: Larger samples receive higher weights, as they provide more accurate estimates to enrich the spatial smoothing function.

### Multilevel analysis

We estimated generalized linear mixed models with households *i* (7281 <= *n* <= 7361) nested in regions *j* (93 <= *n* <= 96) to model the relationship of household-level and regional-level covariates with the probability ${P}({Y}_{{ij}}=1)={\pi }_{{ij}}$ that household *i* in region *j* had a positive response in all relevant survey questions regarding belief = 1, concern = 1, and importance = 1. Consider the simplest form of the model with one household-level predictor *X* (e.g. age) and one regional-level predictor *Z* (e.g. share of green votes in 1994):

5 $$\log \left(\frac{{\pi }_{{ij}}}{1-{\pi }_{{ij}}}\right)={\beta }_{0}+{\beta }_{1}{X}_{{ij}}+{\beta }_{2}{Z}_{j}+{\mu }_{j}$$

${\mu }_{j}$ can be interpreted as the contextual effect of being grouped in *j* on the probability that *Y* = 1. We grouped households into German planning regions, as this level guarantees a sufficiently large number of observations per region (mean *n* = 66.42, min *n* = 5 max *n* = 431). Planning regions are functional units used for large-scale analyses by the federal government for structural purposes such as the distribution of federal funds, projections of population trends, and the assessment of regional disparities in terms of infrastructure or employment. To estimate the relationship between households’ CCOs and regional-level variables, we included a number of household-level that have been shown to predict individual CCOs. All statistical analyses were conducted in R. Multilevel models were estimated using the *glmer()* function included in the *lme4*^80^ package.

#### Household-level variables

We considered control variables at the household level that are associated with individual CCOs as shown by previous research. Gender: Prior research has provided ample evidence that gender is a strong predictor for individual CCOs with women being more aware of climate change than men^20^. Hence, we include a dummy variable reporting the gender (0 = female, 1 = male) of the surveyed head of household. Political Affiliation: Besides gender, political affiliation is a second strong predictor of individual CCOs^5,20^. To include political affiliation in the context of climate change into our models, we used information on households’ preferred political party. In detail, we created a dummy variable that takes the value of 1 if the head of household prefers to vote for the German Green Party (Alliance 90/The Greens) and 0 otherwise. Concern for the environment: Another strong predictor of individual attitudes towards climate change is people’s general concern about the natural environment^20^. People who place more value on protecting the natural environment show higher CCOs than people who tend to place less value on environmental protection. We constructed a dummy variable taking a value of 1 if the head of household is a member of any environmental organization and 0 otherwise to model households’ general concern for the environment. Education: As reported in previous research, education is a positive predictor of individual CCOs^20^. We therefore include a dummy variable equaling 1 if the surveyed head of household attained the highest German school diploma (*Abitur*). Age: Earlier studies have found mixed results and only small effects in the relationship between age and CCOs^20^. We identified an inverted U-shaped relationship between age and households’ CCOs and therefore included age and age squared as control variables. Household Income: Previous studies suggest that income has a positive relationship with CCOs, albeit to a relatively small extent^20^. We include a categorical variable indicating the household’s income. All variables at the household level were collected from the Green Socio-Ecological Panel.

#### Regional-level variables

Based on our analysis in the main text, we included four contextual variables. For the items urban vs. rural and prospering vs. declining we relied on the predefined classification provided by the Federal Institute for Research on Building, Urban Affairs and Spatial Development^68^, which is only available at the municipality level and not the regional level. The region, however, consists of multiple municipalities (except for the federal city-states of Berlin and Hamburg; Bremen, the third federal city-state consists of two municipalities). We therefore calculated the share of people living in an urban municipality to approximate the urbanity of a planning region and the share of people living in a prospering municipality to approximate the prosperity of a planning region. Green: We approximated local green cultures based on vote shares for Alliance 90/The Greens during the general election of 1994. All variables at the regional level were collected from the online database INKAR maintained and provided by the Federal Institute for Research on Building, Urban Affairs and Spatial Development (BBSR). All these variables were accessed in April 2021 at www.inkar.de. Table 3 reports the description and definition of all variables. East: To investigate the strong East-West divide in the current study, we included a dummy variable that equals 1 if people reside in East Germany and 0 otherwise. Berlin was coded as 1 for regressions in the main text. We also provide additional regression results without Berlin in the supplement yielding similar results (see Supplementary Note 12).

**Table 3:** Independent variables included in multilevel regressions, their definition and data sources

Variable	Description	Min	Max	Mean	SD
***Household level*** *(n* = *7361 in final sample)*					
*Gender*	Gender is a dummy variable taking the value 1 if the surveyed head of household is male and 0 if they are female.	0	1	0.65	0.47
*Political Affiliation*	A dummy variable taking the value 1 if the household prefers to vote for the German green party (*Bündnis 90/die Grünen* or Alliance 90/The Greens) and 0 otherwise.	0	1	0.07	0.25
*Environmental Concern*	A dummy variable equaling 1 if surveyed head of household is a member in any nongovernmental environmental organization and 0 otherwise	0	1	0.13	0.34
*Education*	A dummy variable taking the value 1 if the head of household at least attained the general qualification for university entrance (highest German school degree) and 0 otherwise.	0	1	0.30	0.46
*Age*	Individual’s age in years. We also included its squared form in the regression analysis to consider the detected inverted U-shaped relationship between age and a household’s CCOs.	19	88	53.68	13.78
*Income*	A categorical variable ranging from 1 (less than 500 Euro per month) to 12 (more than 5500 Euro per month)	1	12	6.26	2.71
***Regional level*** *(N* *=* *96)*					
*Urban*	The share of people living in an urban municipality within a planning region	9.95	100	49.89	22.61
*Prospering*	The share of people living in a prospering municipality within a planning region	0	100	64.27	28.82
*Green*	The share of votes for the German green party (Alliance 90/The Greens) during the general election in 1994	2.60	12.70	6.56	2.33
*East*	A dummy variables taking the value 1 if the household resides in a region in East Germany or 0 if the household resides in a region in West Germany at the time of the survey. Berlin was coded as 1.	0	1	0.20	0.40

Descriptive statistics and data description of the independent variables of the generalised linear mixed model. The variables at household level are taken from the Green-SOEP^54–59^, the variables at regional level from the INKAR database (www.inkar.de) of the Federal Institute for Research on Building, Urban Affairs and Spatial Development (BBSR).

### Reporting summary

Further information on research design is available in the Nature Portfolio Reporting Summary linked to this article.

### Supplementary information

Supplementary Information

Reporting Summary

## Data Availability

The data that support the findings of this study are available under restricted access for reasons of German data protection from the research data center of RWI—*Leibniz Institute for Economic Research* in Germany. Restrictions apply to the availability of these data, which were used under license for the current study. For this reason, the data cannot be made available in a public repository. The used data is included in the reference list.

## Code availability

Code available on request from the authors.
