# Supplementary material for: Exploring the variances of climate change opinions in Germany at a fine-grained local scale
Source: Nat Commun. 2024 Feb 29;15:1867. doi: 10.1038/s41467-024-45930-8 (PMC10904852; doi:10.1038/s41467-024-45930-8)
Supplement: Supplementary file 1 — Supplementary Information [file 41467_2024_45930_MOESM1_ESM.pdf]

Supplementary Information

Exploring the variances of climate change opinions in Germany at a fine-grained local scale

January 8, 2024

Author list

Lars Mewes[1*], Leonie Tuitjer[2], Peter Dirksmeier[1]

[1] Institute of Economic and Cultural Geography, Leibniz University Hannover, Hannover, Germany.

[2] Artec – Research Center for Sustainability, Bremen University, Bremen, Germany

* Correspondence: mewes@wigeo.uni-hannover.de

Contents

Supplementary Note 1 3
Supplementary Note 2 4
Supplementary Note 3 6
Supplementary Note 4 8
Supplementary Note 5 11
Supplementary Note 6 17
Supplementary Note 7 27
Supplementary Note 8 29
Supplementary Note 9 31
Supplementary Note 10 34
Supplementary Note 11 35
Supplementary Note 12 41
References 43

Supplementary Note 1

The Green Socio-Ecological Panel (Green SOEP): Data generation and sample characteristics

We used individual data from the Green Socio-Ecological Panel (Green SOEP), a nationally representative household survey conducted by the *RWI– Leibniz Institute for Economic Research* and partners between 2012 and 2015.[1–6] Data collection was part of the federally funded project *Evaluating Climate mitigation and Adaptation Strategies (Eval-MAP)* in collaboration with the *Ruhr-University Bochum* und the Mannheim-based *ZEW – Leibniz Centre for European Economic Research*. Eval-MAP was funded by the German *Federal Ministry for Economic Affairs and Climate Action*.

Data collection was conducted in four waves (first wave in October and November 2012 with n = 6,049, second wave in May and June 2013 with n = 6,522, third wave in June 2014 with n = 6,054, and the fourth wave in March and April 2015 with n = 6,389) by *forsa – Society for Social Research and Statistical Analysis* (“*Gesellschaft für Sozialforschung und statistische Analysen*“). Participants were drawn from the *Omninet-Panel* maintained by forsa. Omninet consists of approximately 10,000 German households representative for the German population. Green-SOEP is based on responses by the heads of the surveyed households defined as the person who decides about financial decisions. Respondents were at least 18 years old. Most respondents participated via a programmable online-survey. Households without internet access participated via television through a technical device provided by forsa. By completing the full questionnaire, participants received two bonus points. The bonus points can be exchanged for prizes similar to a payback system. Green SOEP does not redraw underrepresented samples, i.e. each participant is randomly selected. In total, 12,612 distinct households participated in the survey. Response rates were 94.46% (wave 1), 91.34% (wave 2), 91.7% (wave 3), and 90.28%(wave 4).[5,6]

As described in detail in [6], the surveyed households are representative regarding the overall German population of household heads in terms of their regional and socio-economic characteristics. In two thirds of the responses, the head of the surveyed households is male. This share corresponds well with the national census of households. In a validation check, we considered the respondents’ sex to compute regional estimates with very similar results (see Multi-level regression with post-stratification (MRP) below). The survey leans households between 45 and 65 years. We controlled for respondents’ gender and age in the main analysis. The regional sample sizes correlate strongly with the actual population sizes (r = .97). Percentage differences from actual population shares range between -0.31% and 1.38% indicating that there was no significant regional sampling bias. Detailed information about the survey in English is also provided in [6].

Supplementary Note 2

Assessing CCOs using survey data

An opinion reflects a person's view about a specific topic such as climate change.[7] In some cases, an opinion is assessed by asking one question: Which political party do you prefer to vote for? In other cases, the topic under study cannot be assessed by asking just one question, simply because the topic is too complex and involves multiple dimensions. Climate change is such a multi-dimensional topic. Dimensions that are frequently assessed in the CCO literature are, for example, beliefs about the existence and causes of climate change, risk perceptions, or whether and what politics should do against it.[8] Assessing public opinions on climate change therefore requires researchers to rely on multiple survey questions. In research on climate change opinions, however, there is no scientific consensus about the dimensions that reflect a person's opinion on climate change most accurately. In consequence, a heterogeneous set of survey questions with differing rating scales exists in the empirical literature. In their review of 33 articles, Capstick et al. identified 23 different survey questions that are used to assess CCOs.[9]

When examining a person's opinion on climate change, researchers are therefore confronted with selecting specific dimensions of climate change opinions and a set of survey questions to assess them. The selection process can be characterized as a tradeoff between scope and detail. Researchers may increase the level of detail by focusing on a single dimension such as climate change skepticism. To assess skepticism, researchers increase the level of detail by using a comprehensive set of questions[10]. Studies examining skepticism, however, may not cover other dimensions such as concern or support for climate change related policies. In contrast, researchers may be interested in a more general level of public opinion on climate change.[11–14] Hence, they increase the scope by using a broad spectrum of survey question that cover multiple dimensions of climate change while decreasing the level of detail with which the dimensions are assessed.

In this report, we take such a broader perspective on climate change opinions and examine three dimensions: climate change beliefs (do people think that global climate is changing?), concern (are they worried?), and perceived importance for collective response (is the fight against it important?). These three dimensions reflect a person's general opinion on climate change and are considered as key to indicate the level of public awareness concerning climate change. If people believe in the existence of climate change, are concerned, and perceive the fight against it as important, climate action including, for example, mitigation and adaptation policies are more likely to receive greater acceptance in the population.[15]

We cover each dimension with a single survey question. The first dimension reflects a person's belief in the existence of climate change. Belief is frequently examined by asking respondents whether they believe that the world climate is changing. Typical response scales vary between two-point scales (not changing vs. changing) or ordinal scales (definitely not changing, probably not changing, probably changing, definitely changing).[9] Gallup's Poll Social Series (GPSS) adds a temporal dimensions by asking if and when climate change will begin by examining if people think that climate change will never happen, will not happen within own lifetime but will affect future generations, will happen within own lifetime, will happen within a few years, or has already begun.[16] We relied on a similar survey question from the Green SOEP to assess climate change beliefs. In the Green SOEP, households were asked: "The media is reporting about a global climate change. What do you think about it, which of the

following statements do you agree with the most?”. On a four-point scale, possible answers were “climate change will not occur at all” (4.35%), “climate change will take place in the distant future” (4.50%), “climate change will take place in the near future” (9.02%), and “climate change is already taking place” (82.12%). For the main analysis, we transformed the ordinal to a binary response scale. We coded respondents as 1 who answered with “climate change is already taking place” and 0 otherwise. Downgrading response scales from ordinal to binary comes with a loss of information. We therefore conducted additional robustness checks using the original scales for every dimension. These robustness checks indicate that recoding did not affect our main results.

Some studies also assess climate change beliefs by including personal beliefs about the causes of climate change.[9] Such studies therefore use the distinction between the trend and attribution skepticism.[10] Trend skepticism refers to peoples’ belief that the climate is changing. Attribution skepticism examines if people believe that climate change is mainly human made. In our study, we use trend skepticism to assess climate change beliefs. Trend and attribution skepticism are highly correlated in most European countries and Germany more specifically.[17] The high correlation between trend and attribution skepticism suggests that including attribution skepticism would not have added much variance to the assessment of individual climate change opinions.

The second dimension reflects a person’s level of concern. Assessing concern is well-established in the CCO literature.[9] In large public opinion surveys, respondents are typically asked how much they worry about climate change using similar response scales.[16,18] Following the literature, we used a similar survey question from the Green SOEP in which respondents were asked “The media is reporting about a global climate change. Are you concerned about a possible climate change?” On a seven-point scale, potential answers ranged from “not concerned at all” to “very concerned”. For the main analysis, we coded respondents who answered with 5 or higher as 1 (57.69%) and other responses as 0 (42.31%).

The third dimension is a person’s perceived importance to fight climate change. To assess perceived importance, a number of different survey questions is used in the academic literature. Respondents are, for example, asked if they think it is necessary to take steps to reduce the impact of human activities[9], or whether climate change represents the most important problem facing a country.[19] For our analysis, we used the following survey question from the Green SOEP to assess respondents’ perceived importance: “There are a lot of challenges that our society is confronted with. How important is the fight against climate change?”. Possible answers ranged from “totally unimportant” to “very important” (on a five-point scale). For the main analysis, we coded all respondents who answered with 4 and 5 as 1 (84.59%) and all other responses as 0 (15.41%). Our robustness checks reported below indicate that recoding did not affect our results.

For the multilevel regression in the main text, we aggregated the three dimensions to assess respondents’ general opinion on climate change. We coded all respondents with a 1 who believe that climate change is already taking place, are concerned (5 or higher), and perceive the fight against it as important (4 or higher) and 0 otherwise.

Supplementary Note 3

Cross-validation of local CCO estimates: Cross-validating small sample sizes

To cross-validate the accuracy of the spatial smoothing function with actor-based clustering, we followed previous work[12,20] and drew random subsets (n = 99) from the three largest municipalities in Germany (Berlin, Hamburg, Munich) to quantify the effect of small sample sizes on the precision of the spatial smoothing function. The observed mean in the largest municipalities across the full local sample serves as a benchmark. Simulated sample sizes range between 5 and 100. We compared the precision of actor-based clustering with the precision of disaggregation (i.e., simply calculating the average based on n observations in a region). In so doing, we calculated the mean absolute errors (MAEs) of both methods based on the "truly" observed average in the three municipalities.

The cross-validation results are displayed in Supplementary Figure 1 with simulated sample sizes on the x-axis and the mean absolute errors (MAEs) on the y-axis. A MAE of 0.02 is similar to a difference of 2%. MAEs in Supplementary Figure 1 represent the average for the three largest municipalities. Cross-validation indicates that the spatial smoothing function with actor-based clustering (green dots) compared with disaggregation (blue dots) is highly accurate with average MAEs of 1.97% for the three CCO dimensions. The accuracy of actor-based clustering is comparable to methods applied in previous research.[12]

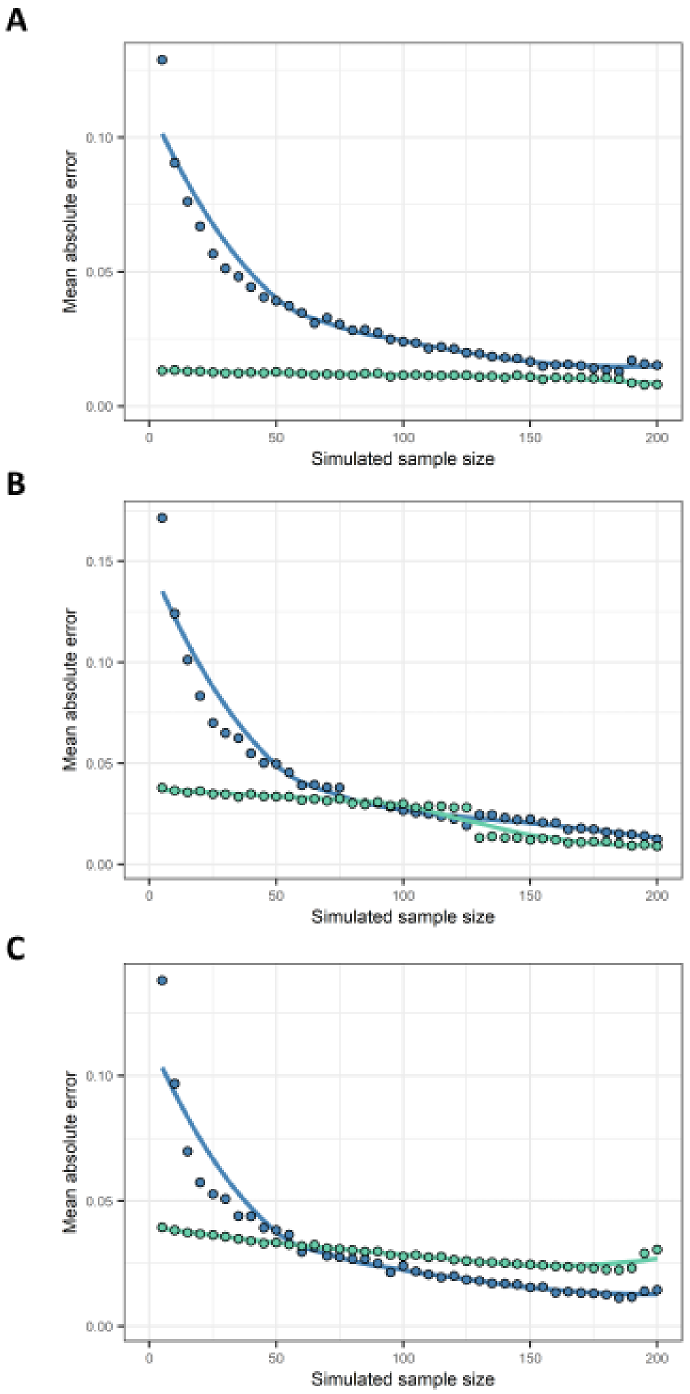

Supplementary Figure 1*: Results of cross-validation for simulated sample sizes (n=99) of actor-based clustering (green dots) and disaggregation (blue dots) for the share of households that belief climate is already taking place* ***(A)****, is very concerned* ***(B)****, and perceives collective response as very important* ***(C)****.*

Supplementary Note 4

Cross-validation of local CCO estimates: Cross-validating the effect of low-populated macro-areas on sparsely populated regions

As actor-based clustering relies on geographic distances and includes observed values in neighboring municipalities by applying spatial weights, the resulting local CCOs rely on observation in the focal municipality as well as on observations in neighboring municipalities. Specifically, less populated regions with small sample sizes located in low-populated macro-areas (many neighbors with equally small population sizes) might therefore suffer from low accuracies in estimated CCOs. We therefore repeated the simulations as described previously by using the three largest municipalities (Berlin, Hamburg, Munich). We held the sample sizes in all three municipalities constant at n=0 to mimic a less populated municipality with a sample size of 0 and drawing random subsets (n=99) from municipalities within a distance of 120km, which is twice the distance of the parameter r as defined in the applied log-logistic distance decay function and therefore represents the distance bound in which spatial weights decrease to nearly 0. The results are depicted in Supplementary Figure 2 indicating constant accuracies for simulated sample weights > 50 for the three CCO dimensions. Sample weights represent the denominator of equation (3) in the main text and are the product of the number of observations (e.g., the number of surveyed households) and the geographic weights. Hence, sample weights do not directly translate into the exact number of observations. They indicate on how much data points the individual CCO values are based: the higher the sample weights, the better the information and the higher the precision of the estimate as shown by our cross-validation (see Supplementary Figure 1). As actor-based clustering produces some inaccuracies (MAEs > 5%) in low-population macros areas (sample weights < 50), we therefore excluded regions with sample weights < 50 (n=96) from the analysis in the main text. This step reduces the number of municipalities from 4,667 to 4,571. In addition, Supplementary Figure 3 shows the distribution of sample weights across German municipalities.

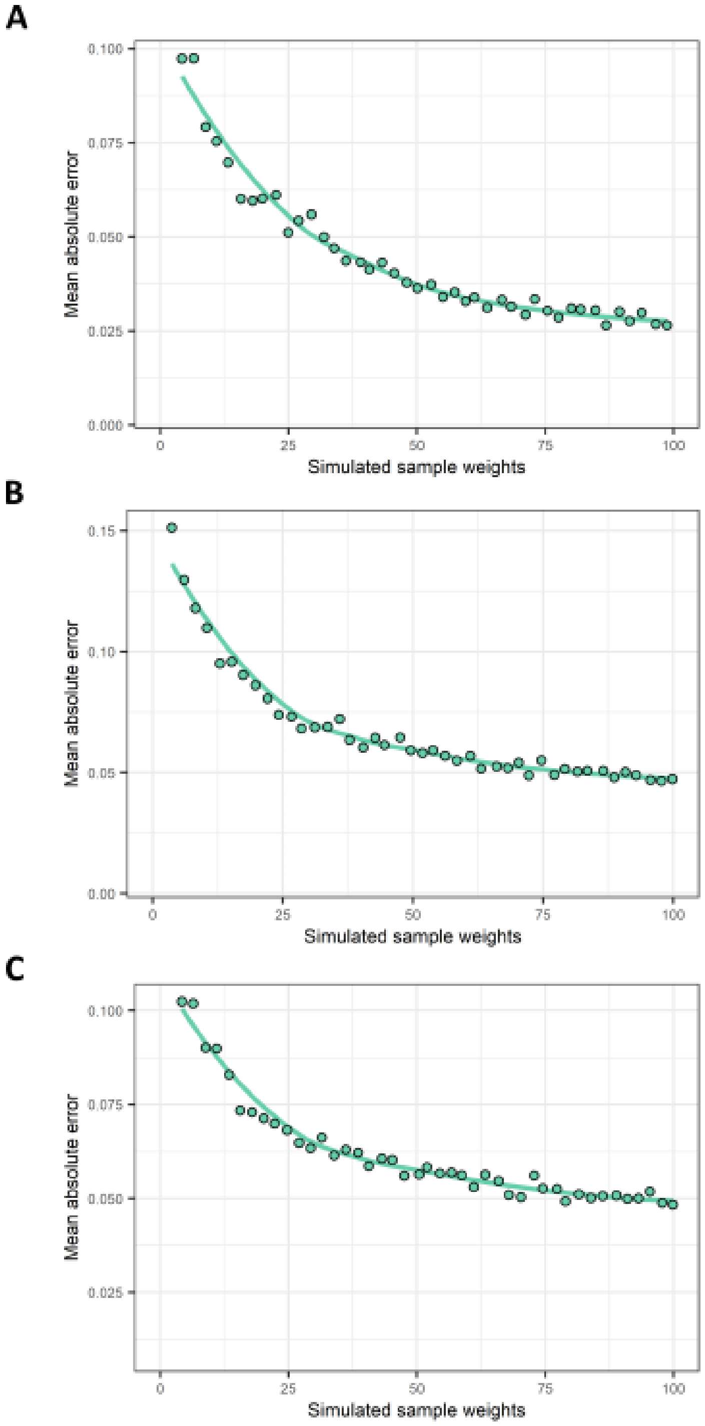

Supplementary Figure 2*: Results of cross-validation for low-population macro areas for simulated sample weights (n=99) of actor-based clustering for the share of households that belief climate is already taking place* ***(A)****, is very concerned* ***(B)****, and perceives collective response as very important* ***(C)****.*

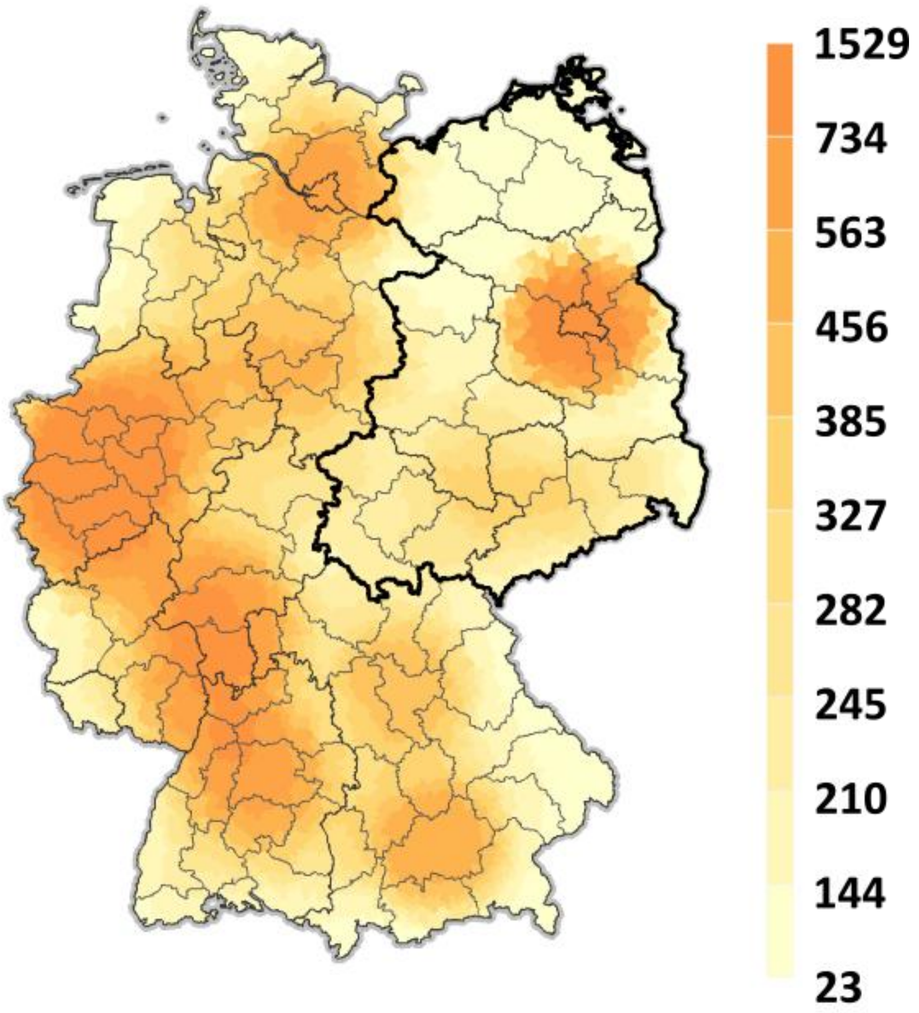

Supplementary Figure 3: *Distribution of sample weights across the 4,667 municipalities in Germany*

Supplementary Note 5

Multi-level regression with post-stratification (MRP)

In the main text, we revealed general geographic patterns using spatial smoothing techniques[21]. Spatial smoothing enabled us to produce geographic maps of public CCOs and to uncover their geographic patterns. Compared with other aggregation techniques such as multilevel regression with post-stratification (MRP), spatial smoothing has a number of advantages. Foremost, this concerns the low entry barriers making spatial smoothing accessible to many researchers. Equipped with a geo-located variable (such as climate change opinions), researchers are able to map a variable on a very fine-grained geographic level such as the 4,667 German municipalities without requiring any additional data or imposing any assumptions on the distribution of the variable.

However, spatial smoothing has been rarely applied in research on public opinion. To evaluate the robustness of the spatial smoothing, we conducted MRP. MRP has been widely used to project public opinions in small spatial areas with small sample sizes.[20,23] Specifically, Howe et al. (2015) applied MRP to reveal geographic variation in public CCOs across US regions. Applying MRP in our context requires four steps[23]. (1) collect survey information on public opinions, (2) estimate a multilevel regression with respondents' CCO as dependent variable, (3) construct a post-stratification frame, and (4) predict and aggregate public opinions to the spatial level of interest based on step (2) and (3). As we will outline below, the construction of the post-stratification frame represents the most critical step in MRP.

Regarding the first step, the Green SOEP provides information on respondents' opinions on climate change and relevant personal characteristics such as respondents' age, their sex, their level of education, or their political preferences. Importantly, the Green SOEP reports respondents' residences allowing us to geo-locate public opinions. In the second step, we estimated a generalized linear multilevel regression with respondents' CCOs defined as a binary variable based on the three given climate change dimensions belief, concern, and importance as dependent variable. The dependent variable is the same that we used in our main regression analysis. As predictors, we included respondents' sex (0 = female, 1 = male), their education (0 = no high-school diploma, 1 = high-school diploma), their age (18 to 30, 31 to 50, 51 to 65, 66 to 75, or 75 and older), and their political preference (0 = not green, 1 = green). These predictors were also used in the main regression analysis except from income (12 income groups) and environmental concern (0 = no membership in environmental non-governmental organization (ENGO), 1 = membership in ENGO). Note that we do not include any predictor at the regional level to facilitate comparison with spatial smoothing.

Ideally, we would have used all predictor variables from the main regression analysis. However, the selection of predictor variables was influenced by the third step – the construction of the so-called *post-stratification frame (PSF).* In contrast to spatial smoothing, MRP has higher requirements regarding data. Specifically, researchers not only require the outcome variable but also the predictor variables (in our case sex, education, age, and political preference) at the same spatial level of interest. The predictors are stored in a PSF that is used in step four to project the individual estimates (obtained in step 2) to the local level. An ideal PSF indicates the joint distribution of the predictors at the local level of interest. Since we predict respondents' CCOs based on their sex (2 groups), age (5 age groups), education (2 groups), and political preferences (2 groups), we require a detailed PSF that indicates the joint distribution of ideal types for every region. In our case, the PSF must ideally indicate the share of females, between 18 and 30, with a high school diploma, and who prefer to vote for the Green party.

Using the independent variables above, one must create a post-stratification frame with 2 times 5 times 2 times 2 (= 40) ideal types.

To finally construct the PSF in the third step, we would ideally have information on the joint distribution of the predictor variables at the spatial level of interest (e.g., the 4,667 municipalities). Data availability, however, limited the construction of the PSF in two ways. First, detailed information on the joint distribution of the predictor variables at a fine-grained geographic level is in our case not available. We therefore constructed the PSF by using information on the marginal distribution of variables.[23] The second limitation concerns the geographic level for which the PSF is constructed. Constructing the PSF at the level of 4,667 municipalities was not possible, because secondary data for all predictor variables was not available at such a detailed geographic level. We therefore constructed the MRP at the level of the 96 planning regions ("*Raumordnungsregionen*"). Spatial planning regions are functionally delimited statistical spatial units based on core-surrounding relationships that form the basis of federal spatial planning. Using marginal distributions at the level of planning regions represented the only opportunity to conduct MRP in our case.

In the fourth step, we used the PSF (step three) and the point estimates obtained from the multilevel model (step two) to project the share of people who belief in climate change, are concerned, and regard collective action against it as important to the regional level. Technically, we conducted MRP in R using the *autoMrP* package.[24]

Remember that we applied MRP and spatial smoothing at different spatial levels. To compare the results of the MRP with those obtained by spatial smoothing, we aggregated the results obtained by spatial smoothing to the level of the 96 planning regions. Supplementary Figure 4 depicts the final results in geographic maps. The geographic patterns of CCOs produced by MRP (mean = 52.59%, min = 40.52%, max = 63.17%, sd = 5.12%) are similar to those obtained by spatial smoothing (mean = 56.98%, min = 42.83%, max = 67.13%, sd = 4.48%). The correlation coefficient between MRP and spatial smoothing is 0.7 ($p < 0.001$) (see Supplementary Figure 5 for scatterplot) and the mean absolute error is 4.8%. Supplementary Figure 6 depicts 95% confidence intervals of regional MRP estimates based on bootstrapping.

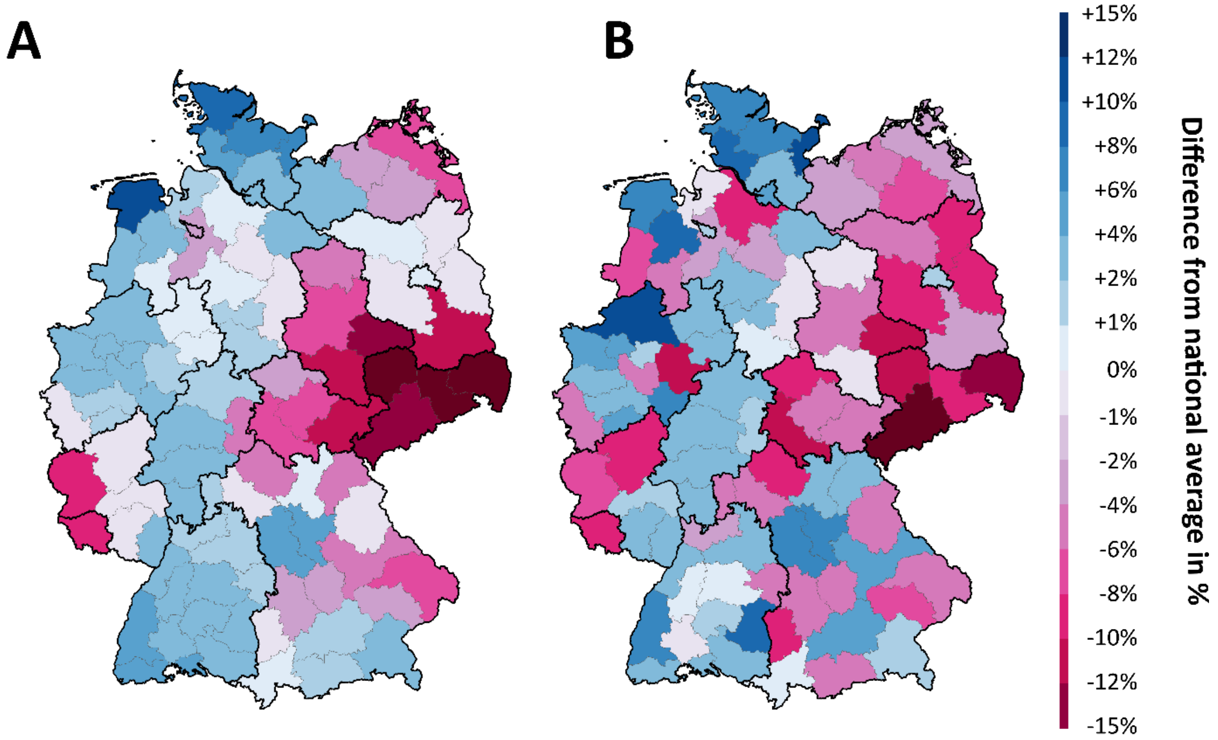

Supplementary Figure 4: *Regional estimates of public at the level of 96 German planning regions (borders are depicted in light grey) based on **(A)** spatial smoothing and **(B)** MRP. The maps indicate the percentage difference from the national average. Solid black lines depict the 16 federal states.*

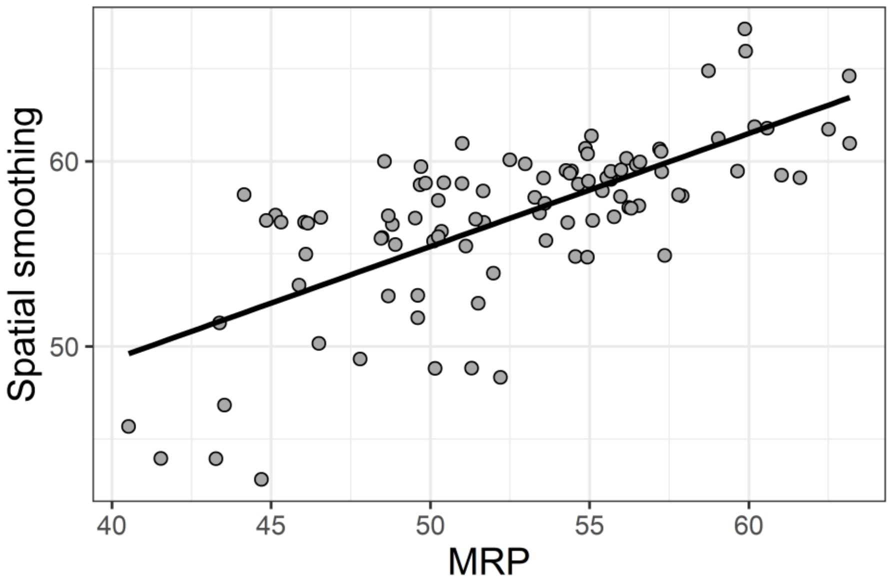

Supplementary Figure 5: *Regional estimates of public CCOs based on MRP and spatial smoothing at the level of 96 German planning regions. Correlation coefficient is 0.7 with p < 0.001.*

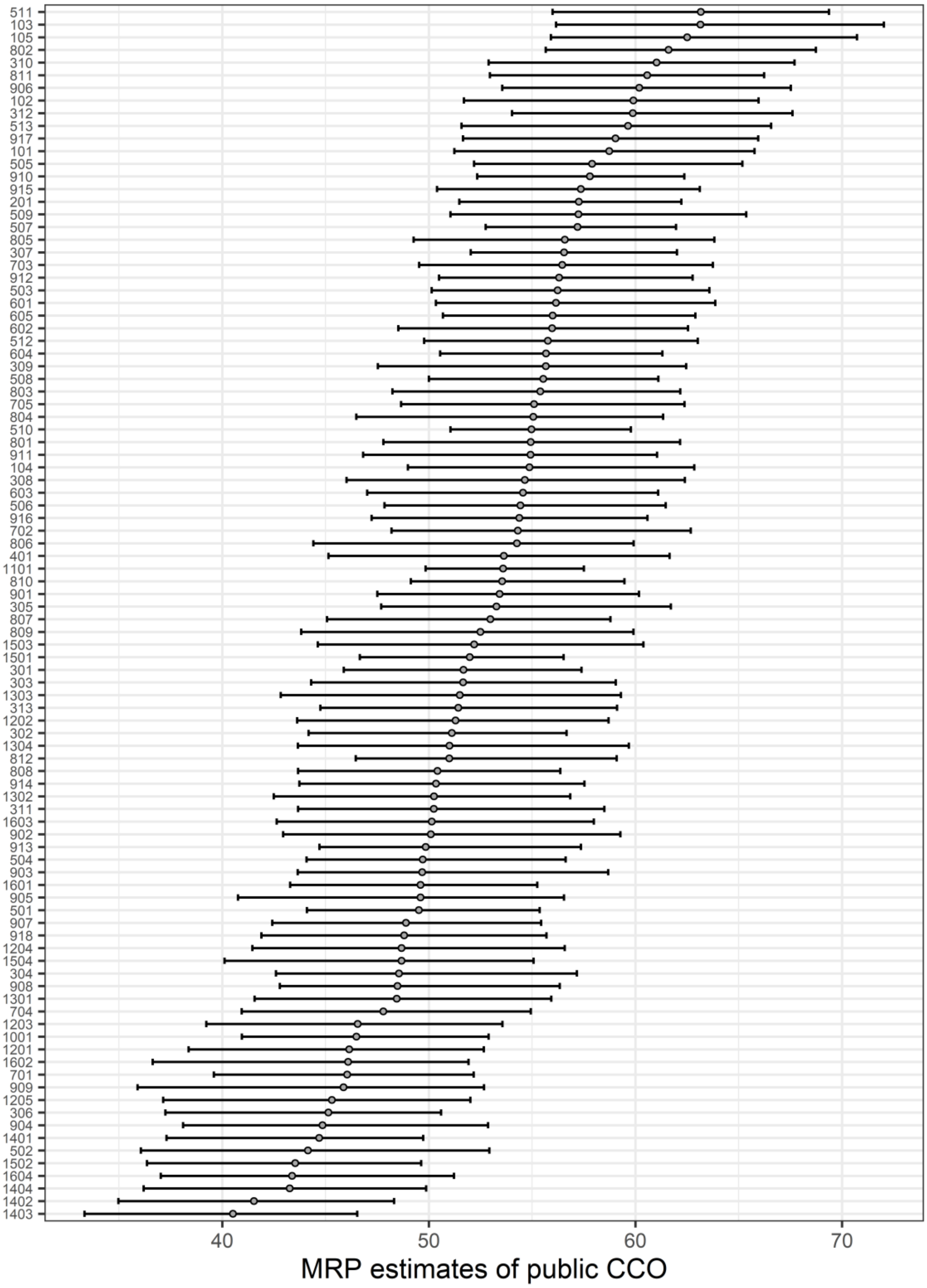

Supplementary Figure 6*: Regional estimates of MRP and their 95% confidence intervals based on bootstrapping at the level of 96 German planning regions (regional identifiers on y-axis).*

To further validate our main findings, we evaluated how the MRP results relate to the four geographic features (urban, prospering, East, green). In the main text, we relied on the official classification provided by the *Federal Institute for Research on Building, Urban Affairs and Spatial Development (BBSR)*, which is only available at the municipality level and not the regional level. The region, however, consists of multiple municipalities (except for the federal city-states of Berlin and Hamburg. Bremen,

the third federal city state consists of two municipalities). We therefore calculated the share of people living in an urban municipality to approximate the urbanity of a planning region and the share of people living in a prospering municipality to approximate the prosperity of a planning region. We approximated local green cultures based on vote shares for Alliance 90/The Greens during the general election of 1994. All these variables at the regional level were collected from the online database *INKAR* maintained and provided by the *BBSR.* To differentiate between East and West, we created a dummy variable that is 1 in case of Eastern regions and 0 otherwise.

Since urban, prospering, and green represent percentages, we estimated their relationship with regional CCOs using bivariate correlations (see Supplementary Figure 7 and Supplementary Table 1). MRP and spatial smoothing produce very similar results. The relationship between CCOs and urban is positive but results are not robust regarding statistical significance at the 5% level. The shares of prospering and green votes correlate positively with CCOs in regions. Lastly, the East-West differences as reported in the main text are substantiated by the MRP results. Since, the East dummy is categorical, we computed a two-sided t-test to validate the East-West differences. The average share of people, who believe in climate change, is concerned, and perceives the fight against it as important in the East of Germany, (47.66%) is significantly different ($t = 6.67$, $p < 0.001$) from the share in Western (54.05%) regions.

In sum, we estimated regional CCOs based on two fundamentally different methodologies. Both methodologies, MRP and spatial smoothing, produced very similar results. MRP therefore validated the findings from the main text obtained by spatial smoothing. We interpret these robustness checks as substantial empirical evidence that the revealed geographic patterns in the main text are not an artefact of the spatial smoothing approach, but instead represent existing regional differences in public CCOs.

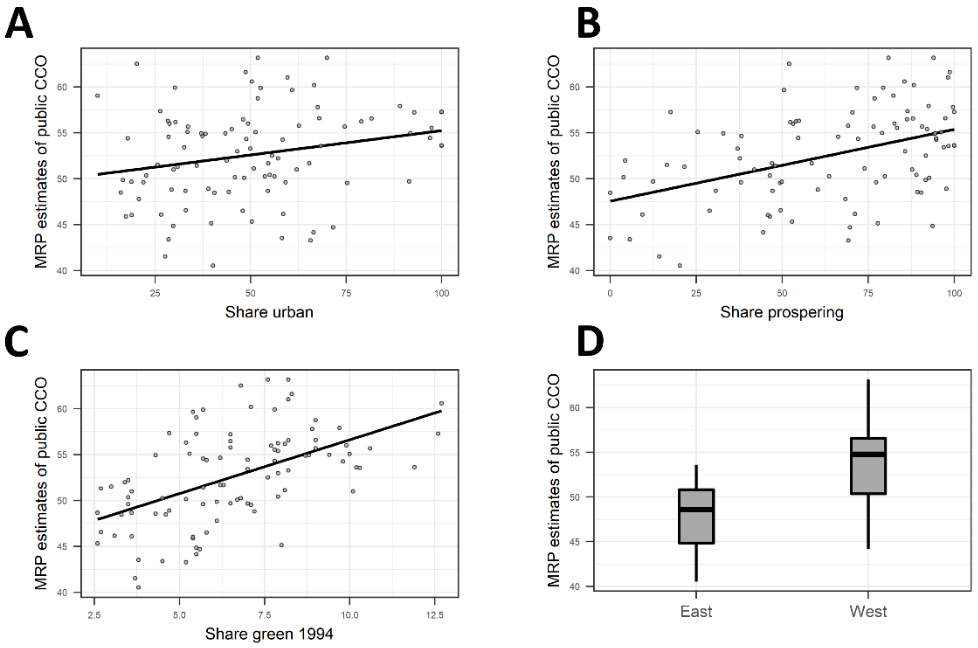

Supplementary Figure 7*: Regional estimates of public CCOs based on MRP at the level of 96 planning regions. Panels* ***A-D*** *depict the relationship between regional CCOs to the four geographic features of* ***(A)*** *urban vs. rural,* ***(B)*** *prospering vs. declining,* ***(C)*** *green vs. non-green, and* ***(D)*** *East vs. West. Note that the difference between East and West German municipalities regarding their MRP estimates has been summarized in a standard Box Plot. The central horizontal lines in each box represent the groupwise median. The lower and upper bounds of the box correspond to the first and third quartiles (i.e., the 25th and 75th percentiles). The upper and lower whisker extend from the lower and upper bounds of the box no further than 1.5 times the inter-quartile range (IQR).*

Supplementary Table1: *Comparison of correlation coefficients (r) with corresponding p-values (p) and 95% confidence intervals (CIs) of MRP and spatial smoothing*

	MRP<br>r (p)<br>[95% CI]	Spatial Smoothing<br>r (p)<br>[95% CI]
Urban	0.23 (0.02)<br>[0.04; 0.41]	0.14 (0.19)<br>[-0.07; 0.33]
Prospering	0.44 (<0.001)<br>[0.26; 0.59]	0.48 (<0.001)<br>[0.31; 0.62]
Green	0.53 (<0.001)<br>[0.37; 0.66]	0.55 (<0.001)<br>[0.39; 0.68]

Supplementary Note 6

Each CCO dimension as outcome variable

In the main regression analysis, we used aggregated opinions as the dependent variables. To test whether aggregation affected our results, we used each CCO dimension (belief, concern, importance) as the outcome and re-estimated the generalized linear multilevel models. Results are reported in Supplementary Tables 2, 3, and 4. Despite some minor changes in the point estimates of the individual level variables, the results of the context-level variables are robust throughout all the specifications. Hence, aggregation did not affect our main regression results.

Supplementary Table 2: *Regression results for belief in climate change as dependent variable*

	Urban vs. Rural	Prospering vs. Declining	Green vs. Non-green	East vs. West
	exp(β) (P) [95% CI]	exp(β) (P) [95% CI]	exp(β) (P) [95% CI]	exp(β) (P) [95% CI]
Household level				
(Intercept)	4.44 (<0.001)	4.43 (<0.001)	4.35 (<0.001)	4.93 (<0.001)
	[3.72; 5.30]	[3.73; 5.27]	[3.66; 5.18]	[4.13; 5.88]
Sex (1=Male)	0.80 (0.001)	0.80 (0.001)	0.80 (0.001)	0.79 (<0.001)
	[0.70; 0.91]	[0.70; 0.91]	[0.70; 0.92]	[0.69; 0.90]
Age	2.80 (<0.001)	2.91 (<0.001)	2.88 (<0.001)	2.78) (<0.001)
	[1.85; 4.22]	[1.93; 4.38]	[1.91; 4.35]	[1.84; 4.19]
Age (Squared)	0.30 (<0.001)	0.29 (<0.001)	0.29 (<0.001)	0.31 (<0.001)
	[0.20; 0.45]	[0.20; 0.44]	[0.20; 0.44]	[0.20; 0.46]
Education	1.42 (<0.001)	1.43 (<0.001)	1.42 (<0.001)	1.46 (<0.001)
	[1.22; 1.65]	[1.23; 1.66]	[1.23; 1.65]	[1.26; 1.70]
Income	0.99 (0.253)	0.98 (0.175)	0.98 (0.209)	0.98 (0.117)
	[0.96; 1.01]	[0.96; 1.01]	[0.96; 1.01]	[0.96; 1.00]
Political Affiliation	3.45 (<0.001)	3.40 (<0.001)	3.40 (<0.001)	3.41 (<0.001)
(1=Green Party)	[2.34; 5.10]	[2.30; 5.03]	[2.30; 5.02]	[2.31; 5.03]
Environmental Concern	1.64 (<0.001)	1.63 (<0.001)	1.63 (<0.001)	1.61 (<0.001)
	[1.34; 2.02]	[1.32; 2.00]	[1.33; 2.00]	[1.31; 1.98]
Regional level				
Urban	1.01 (0.739)			
	[0.95; 1.08]			
Prospering		1.13 (0.001)		
		[1.05; 1.21]		
Green			1.10 (0.004)	
			[1.03; 1.18]	
East				0.71 (<0.001)
				[0.61; 0.82]
AIC	7,013.15	7,001.80	7,005.30	6,996.17
BIC	7,082.19	7,070.84	7,074.34	7,065.21
R^2 (conditional)	0.082	0.083	0.082	0.081
R^2 (marginal)	0.075	0.078	0.077	0.080
N individuals	7,361	7,361	7,361	7,361
N regions	96	96	96	96
Variance (regions)	0.03	0.02	0.02	0.00

Binary logistic multilevel models with households nested in German planning regions. *Dependent variable (CCO) equals 1 if household believes in climate change and 0 otherwise.* 95% confidence intervals in square brackets. See Methods section in the main text for a detailed description of all variables included in the model. All continuous variables were z-standardized beforehand.

Supplementary Table 3: Regression results for concern about climate change as dependent variable

	Urban vs. Rural	Prospering vs. Declining	Green vs. Non-green	East vs. West
	exp(β) (P) [95% CI]	exp(β) (P) [95% CI]	exp(β) (P) [95% CI]	exp(β) (P) [95% CI]
Household level				
(Intercept)	1.50 (<0.001)	1.52 (<0.001)	1.50 (<0.001)	1.67 (<0.001)
	[1.31; 1.71]	[1.33; 1.73]	[1.31; 1.71]	[1.46; 1.92]
Sex (1=Male)	0.81 (<0.001)	0.81 (<0.001)	0.81 (<0.001)	0.80 (<0.001)
	[0.73; 0.90]	[0.73; 0.90]	[0.73; 0.90]	[0.72; 0.88]
Age	1.33 (0.094)	1.37 (0.066)	1.37 (0.065)	1.33 (0.096
	[0.95; 1.86]	[0.98; 1.91]	[0.98; 1.92]	[0.95; 1.86]
Age (Squared)	0.78 (0.147)	0.76 (0.109)	0.76 (0.106)	0.78 (0.150)
	[0.56; 1.09]	[0.55; 1.06]	[0.54; 1.06]	[0.56; 1.09]
Education	1.09 (0.148)	1.10 (0.104)	1.09 (0.123)	1.12 (0.048)
	[0.97; 1.22]	[0.98; 1.23]	[0.98; 1.22]	[1.00; 1.26]
Income	0.98 (0.071)	0.98 (0.039)	0.98 (0.045)	0.98 (0.017)
	[0.96; 1.00]	[0.96; 1.00]	[0.96; 1.00]	[0.96; 1.00]
Political Affiliation	3.28 (<0.001)	3.26 (<0.001)	3.24 (<0.001)	3.25 (<0.001)
(1=Green Party)	[2.58; 4.18]	[2.56; 4.15]	[2.55; 4.13]	[2.55; 4.14]
Environmental Concern	1.83 (<0.001)	1.81 (<0.001)	1.82 (<0.001)	1.80 (<0.001)
	[1.57; 2.13]	[1.56; 2.11]	[1.56; 2.12]	[1.55; 2.10]
Regional level				
Urban	1.05 (0.065)			
	[1.00; 1.10]			
Prospering		1.09 (0.001)		
		[1.04; 1.15]		
Green			1.09 (0.001)	
			[1.03; 1.15]	
East				0.76 (<0.001)
				[0.67; 0.87]
AIC	9,761.67	9,754.52	9,754.49	9,747.41
BIC	9,830.71	9,823.56	9,823.53	9,816.45
R^2 (conditional)	0.055	0.054	0.055	0.055
R^2 (marginal)	0.052	0.054	0.054	0.055
N individuals	7,361	7,361	7,361	7,361
N regions	96	96	96	96
Variance (regions)	0.01	0.00	0.00	0.00

Binary logistic multilevel models with households nested in German planning regions. *Dependent variable (CCO) equals 1 if household is concerned about climate change and 0 otherwise.* Coefficients represent odd ratios. 95% confidence intervals in square brackets. See Methods section in the main text for a detailed description of all variables included in the model. All continuous variables were z-standardized beforehand.

Supplementary Table 4: *Regression results for importance of collective response to fight climate change as dependent variable*

	**Urban vs. Rural**	**Prospering vs. Declining**	**Green vs. Non-green**	**East vs. West**
	exp(*β*) (P) [95% CI]	exp(*β*) (P) [95% CI]	exp(*β*) (P) [95% CI]	exp(*β*) (P) [95% CI]
Household level				
(Intercept)	4.44 (<0.001)	4.43 (<0.001)	4.35 (<0.001)	4.93 (<0.001)
	[3.72; 5.30]	[3.73; 5.27]	[3.66; 5.18]	[4.13; 5.88]
Sex (1=Male)	0.80 (<0.001)	0.80 (<0.001)	0.80 (<0.001)	0.79 (<0.001)
	[0.70; 0.91]	[0.70; 0.91]	[0.70; 0.92]	[0.69; 0.90]
Age	2.80 (0.010)	2.91 (0.006)	2.88 (0.006)	2.78 (0.009)
	[1.85; 4.22]	[1.93; 4.38]	[1.91; 4.35]	[1.84; 4.19]
Age (Squared)	0.30 (0.048)	0.29 (0.032)	0.29 (0.029)	0.31 (0.042)
	[0.20; 0.45]	[0.20; 0.44]	[0.20; 0.44]	[0.20; 0.46]
Education	1.42 (<0.001)	1.43 (<0.001)	1.42 (<0.001)	1.46 (<0.001)
	[1.22; 1.65]	[1.23; 1.66]	[1.23; 1.65]	[1.26; 1.70]
Income	0.99 (0.733)	0.98 (0.897)	0.98 (0.816)	0.98 (0.859)
	[0.96; 1.01]	[0.96; 1.01]	[0.96; 1.01]	[0.96; 1.00]
Political Affiliation	3.45 (<0.001)	3.40 (<0.001)	3.40 (<0.001)	3.41 (<0.001)
(1=Green Party)	[2.34; 5.10]	[2.30; 5.03]	[2.30; 5.02]	[2.31; 5.03]
Environmental Concern	1.64 (<0.001)	1.63 (<0.001)	1.63 (<0.001)	1.61 (<0.001)
	[1.34; 2.02]	[1.32; 2.00]	[1.33; 2.00]	[1.31; 1.98]
Regional level				
Urban	1.01 (0.539)			
	[0.95; 1.08]			
Prospering		1.13 (<0.001)		
		[1.05; 1.21]		
Green			1.10 (<0.001)	
			[1.03; 1.18]	
East				0.71 (<0.001)
				[0.61; 0.82]
AIC	5,797.15	5,784.75	5,782.07	5,771.45
BIC	5,866.19	5,853.79	5,851.11	5,840.48
R^2 (conditional)	0.121	0.121	0.120	0.124
R^2 (marginal)	0.105	0.112	0.113	0.116
N individuals	7,361	7,361	7,361	7,361
N regions	96	96	96	96
Variance (regions)	0.06	0.04	0.03	0.03

Binary logistic multilevel models with households nested in German planning regions. *Dependent variable (CCO) equals 1 if household perceives collective response to fight climate change as important and 0 otherwise*. Coefficients represent odd ratios. 95% confidence intervals in square brackets. See Methods section in the main text for a detailed description of all variables included in the model. All continuous variables were z-standardized beforehand.

Using the original ordinal response scales

Note that we used a binary coding scheme for the main analysis. Using binary outcomes is well-established in the literature on CCOs to simply report shares of the population that, for example, believe climate change has already begun[11], to predict respondents' responses using generalized linear multilevel regressions[17], or to project regional estimates using multilevel with post-stratification (MRP)[12]. These three reasons all apply for our analysis, since we produced geographic maps showing regional shares, predicted responses using generalized linear multilevel regressions, and projected local level estimates using MRP. However, downgrading scales from ordinal to binary can be problematic due to the loss of information. Although linear modeling on ordinal scales is also not an ideal solution, we re-estimated the models from the main text using the original response scales using linear multilevel models. Despite some minor fluctuations of estimates for some individual-level variables (i.e., income and education), the results remained robust for the majority of variables including the context-level variables (see Supplementary Tables 5, 6, and 7). Hence this robustness check indicates that recoding did not affect our estimations results.

Supplementary Table 5: *Regression results of linear multilevel model with climate change belief as dependent variable*

	**Without local context**	**Urban vs. Rural**	**Prospering vs. Declining**	**Green vs. Non-green**	**Green individual vs. Green regional**	**East vs. West**
	β (P) [95% CI]	*β* (P) [95% CI]	*β* (P) [95% CI]	*β* (P) [95% CI]	*β* (P) [95% CI]	*β* (P) [95% CI]
Household level						
(Intercept)	3.70 (<0.001) [3.65; 3.75]	3.70 (<0.001) [3.65; 3.75]	3.70 (<0.001) [3.65; 3.75]	3.69 (<0.001) [3.64; 3.74]	3.69 (<0.001) [3.64; 3.74]	3.73 (<0.001) [3.68; 3.78]
Sex (1=Male)	-0.09 (<0.001) [-0.13; -0.06]	-0.09 (<0.001) [-0.13; -0.06]	-0.09 (<0.001) [-0.13; -0.06]	-0.09 (<0.001) [-0.13; -0.05]	-0.09 (<0.001) [-0.13; -0.05]	-0.10 (<0.001) [-0.14; -0.06]
Age	0.29 (<0.001) [0.17; 0.41]	0.29 (<0.001) [0.17; 0.42]	0.30 (<0.001) [0.18; 0.43]	0.30 (<0.001) [0.18; 0.43]	0.30 (<0.001) [0.18; 0.43]	0.29 (<0.001) [0.17; 0.41]
Age (Squared)	-0.32 (<0.001) [-0.44; -0.19]	-0.32 (<0.001) [-0.44; -0.19]	-0.33 (<0.001) [-0.45; -0.20]	-0.33 (<0.001) [-0.45; -0.20]	-0.33 (<0.001) [-0.45; -0.20]	-0.32 (<0.001) [-0.44; -0.19]
Education (1= High School)	0.09 (<0.001) [0.05; 0.13]	0.09 (<0.001) [0.05; 0.13]	0.09 (<0.001) [0.05; 0.13]	0.09 (<0.001) [0.05; 0.13]	0.09 (<0.001) [0.05; 0.13]	0.10 (<0.001) [0.06; 0.14]
Income	-0.00 (0.194) [-0.01; 0.00]	-0.00 (0.201) [-0.01; 0.00]	-0.01 (0.136) [-0.01; 0.00]	-0.00 (0.166) [-0.01; 0.00]	-0.00 (0.166) [-0.01; 0.00]	-0.01 (0.092) [-0.01; 0.00]
Political Affiliation (1=Green Party)	0.20 (<0.001) [0.13; 0.27]	0.20 (<0.001) [0.13; 0.27]	0.20 (<0.001) [0.13; 0.27]	0.20 (<0.001) [0.13; 0.27]	0.22 (<0.001) [0.14; 0.30]	0.19 (<0.001) [0.12; 0.27]
Environmental Concern	0.12 (<0.001) [0.06; 0.17]	0.12 (<0.001) [0.06; 0.17]	0.11 (<0.001) [0.06; 0.16]	0.11 (<0.001) [0.06; 0.17]	0.11 (<0.001) [0.06; 0.17]	0.11 (<0.001) [0.06; 0.16]
Regional level						
Urban		0.01 (0.421) [-0.01; 0.03]				
Prospering			0.04 (<0.001) [0.02; 0.06]			
Green				0.03 (0.003) [0.01; 0.05]	0.03 (0.002) [0.01; 0.05]	
East						-0.11 (<0.001) [-0.15; -0.06]
Cross-level interaction						
Political Affiliation x Green					-0.04 (0.312) [-0.11; 0.04]	
AIC	16,953.13	16,961.92	16,948.96	16,952.99	16,958.73	16,942.19
BIC	17,022.17	17,037.86	17,024.90	17,028.94	17,041.58	17,018.13
R^2 (conditional)	0.024	0.025	0.026	0.025	0.025	0.025
R^2 (marginal)	0.021	0.021	0.023	0.023	0.023	0.024
n individuals	7,361	7,361	7,361	7,361	7,361	7,361
n regions	96	96	96	96	96	96
Variance (regions)	0.00	0.00	0.00	0.00	0.00	0.00

Linear multilevel models with households nested in German planning regions. *Dependent variable is climate change belief measured on a four-point scale ranging between 1 "climate change will not occur at all" and 4 "climate change is already taking place".* All continuous variables were z-standardized beforehand.

Supplementary Table 6: *Regression results of linear multilevel model with climate change concern as dependent variable*

	**Without local context**	**Urban vs. Rural**	**Prospering vs. Declining**	**Green vs. Non-green**	**Green individual vs. Green regional**	**East vs. West**
	β (P) [95% CI]	*β* (P) [95% CI]	*β* (P) [95% CI]	*β* (P) [95% CI]	*β* (P) [95% CI]	*β* (P) [95% CI]
Household level						
(Intercept)	4.78 (<0.001) [4.68; 4.89]	4.77 (<0.001) [4.66; 4.88]	4.78 (<0.001) [4.67; 4.88]	4.76 (<0.001) [4.66; 4.86]	4.76 (<0.001) [4.65; 4.86]	4.87 (<0.001) [4.76; 4.98]
Sex (1=Male)	-0.28 (<0.001) [-0.36; -0.20]	-0.28 (<0.001) [-0.36; -0.20]	-0.28 (<0.001) [-0.36; -0.20]	-0.27 (<0.001) [-0.36; -0.19]	-0.27 (<0.001) [-0.35; -0.19]	-0.29 (<0.001) [-0.37; -0.21]
Age	0.40 (0.004) [0.13; 0.66]	0.40 (0.003) [0.13; 0.67]	0.42 (0.002) [0.16; 0.69]	0.43 (0.002) [0.16; 0.70]	0.43 (0.001) [0.17; 0.70]	0.40 (0.004) [0.13; 0.66]
Age (Squared)	-0.28 (0.039) [-0.54; -0.01]	-0.28 (0.036) [-0.55; -0.02]	-0.31 (0.024) [-0.57; -0.04]	-0.32 (0.020) [-0.58; -0.05]	-0.32 (0.019) [-0.58; -0.05]	-0.28 (0.039) [-0.54; -0.01]
Education (1= High School)	0.03 (0.507) [-0.06; 0.12]	0.03 (0.576) [-0.06; 0.12]	0.03 (0.484) [-0.06; 0.12]	0.03 (0.568) [-0.06; 0.12]	0.03 (0.573) [-0.06; 0.11]	0.05 (0.269) [-0.04; 0.14]
Income	-0.02 (0.009) [-0.03; -0.01]	-0.02 (0.009) [-0.03; -0.00]	-0.02 (0.005) [-0.04; -0.01]	-0.02 (0.006) [-0.04; -0.01]	-0.02 (0.006) [-0.04; -0.01]	-0.02 (0.001) [-0.04; -0.01]
Political Affiliation (1=Green Party)	0.84 (<0.001) [0.68; 0.99]	0.83 (<0.001) [0.68; 0.99]	0.83 (<0.001) [0.68; 0.98]	0.82 (<0.001) [0.67; 0.97]	0.89 (<0.001) [0.71; 1.07]	0.82 (<0.001) [0.67; 0.97]
Environmental Concern	0.51 (<0.001) [0.39; 0.62]	0.51 (<0.001) [0.39; 0.62]	0.50 (<0.001) [0.39; 0.61]	0.50 (<0.001) [0.39; 0.61]	0.50 (<0.001) [0.39; 0.61]	0.49 (<0.001) [0.38; 0.60]
Regional level						
Urban		0.03 (0.166) [-0.01; 0.07]				
Prospering			0.08 (<0.001) [0.04; 0.12]			
Green				0.08 (<0.001) [0.04; 0.12]	0.09 (<0.001) [0.05; 0.13]	
East						-0.25 (<0.001) [-0.34; -0.15]
Cross-level interaction						
Political Affiliation x Green					-0.11 (0.154) [-0.27; 0.04]	
AIC	28,240.91	28,246.95	28,235.44	28,231.57	28,234.76	28,222.69
BIC	28,309.95	28,322.89	28,311.38	28,307.52	28,317.61	28,298.63
R^2 (conditional)	0.047	0.047	0.047	0.048	0.048	0.048
R^2 (marginal)	0.045	0.045	0.047	0.047	0.048	0.048
n individuals	7,361	7,361	7,361	7,361	7,361	7,361
n regions	96	96	96	96	96	96
Variance (regions)	0.01	0.01	0.00	0.00	0.00	0.00

Linear multilevel models with households nested in German planning regions. *Dependent variable is climate change concern measured on a seven-point scale ranging between 1 "not concerned at all" and 7 "very concerned".* All continuous variables were z-standardized beforehand.

Supplementary Table 7: *Regression results of linear multilevel model with perceived importance of climate change as dependent variable*

	**Without local context**	**Urban vs. Rural**	**Prospering vs. Declining**	**Green vs. Non-green**	**Green individual vs. Green regional**	**East vs. West**
	β (P) [95% CI]	*β* (P) [95% CI]	*β* (P) [95% CI]	*β* (P) [95% CI]	*β* (P) [95% CI]	*β* (P) [95% CI]
Household level						
(Intercept)	4.51 (<0.001) [4.45; 4.57]	4.51 (<0.001) [4.45; 4.56]	4.51 (<0.001) [4.45; 4.56]	4.50 (<0.001) [4.44; 4.56]	4.50 (<0.001) [4.44; 4.55]	4.56 (<0.001) [4.50; 4.62]
Sex (1=Male)	-0.22 (<0.001) [-0.26; -0.18]	-0.22 (<0.001) [-0.26; -0.18]	-0.22 (<0.001) [-0.26; -0.18]	-0.22 (<0.001) [-0.26; -0.17]	-0.22 (<0.001) [-0.26; -0.17]	-0.22 (<0.001) [-0.26; -0.18]
Age	0.29 (<0.001) [0.15; 0.43]	0.29 (<0.001) [0.15; 0.43]	0.30 (<0.001) [0.16; 0.44]	0.30 (<0.001) [0.16; 0.44]	0.30 (<0.001) [0.17; 0.44]	0.29 (<0.001) [0.15; 0.43]
Age (Squared)	-0.23 (0.001) [-0.37; -0.09]	-0.23 (<0.001) [-0.37; -0.09]	-0.24 (<0.001) [-0.38; -0.10]	-0.25 (<0.001) [-0.38; -0.11]	-0.25 (<0.001) [-0.38; -0.11]	-0.23 (<0.001) [-0.37; -0.10]
Education (1= High School)	-0.10 (<0.001) [-0.15; -0.05]	-0.10 (<0.001) [-0.15; -0.05]	-0.10 (<0.001) [-0.14; -0.05]	-0.10 (<0.001) [-0.15; -0.05]	-0.10 (<0.001) [-0.15; -0.05]	-0.09 (<0.001) [-0.14; -0.04]
Income	-0.01 (0.176) [-0.01; 0.00]	-0.01 (0.179) [-0.01; 0.00]	-0.01 (0.122) [-0.01; 0.00]	-0.01 (0.141) [-0.01; 0.00]	-0.01 (0.141) [-0.01; 0.00]	-0.01 (0.064) [-0.02; 0.00]
Political Affiliation (1=Green Party)	0.37 (<0.001) [0.29; 0.45]	0.37 (<0.001) [0.29; 0.45]	0.37 (<0.001) [0.29; 0.45]	0.37 (<0.001) [0.29; 0.44]	0.39 (<0.001) [0.30; 0.49]	0.36 (<0.001) [0.29; 0.44]
Environmental Concern	0.19 (<0.001) [0.13; 0.25]	0.19 (<0.001) [0.13; 0.25]	0.19 (<0.001) [0.13; 0.24]	0.19 (<0.001) [0.13; 0.25]	0.19 (<0.001) [0.13; 0.25]	0.18 (<0.001) [0.13; 0.24]
Regional level						
Urban		0.01 (0.528) [-0.02; 0.03]				
Prospering			0.05 (<0.001) [0.02; 0.08]			
Green				0.05 (<0.001) [0.03; 0.08]	0.06 (<0.001) [0.03; 0.08]	
East						-0.18 (<0.001) [-0.24; -0.12]
Cross-level interaction						
Political Affiliation x Green					-0.04 (0.304) [-0.12; 0.04]	
AIC	18,586.76	18,595.24	18,581.59	18,578.20	18,583.67	18,561.84
BIC	18,655.80	18,671.18	18,657.54	18,654.14	18,666.52	18,637.78
R^2 (conditional)	0.053	0.054	0.054	0.054	0.054	0.056
R^2 (marginal)	0.045	0.045	0.048	0.049	0.049	0.052
n individuals	7,361	7,361	7,361	7,361	7,361	7,361
n regions	96	96	96	96	96	96
Variance (regions)	0.01	0.01	0.00	0.00	0.00	0.00

Linear multilevel models with households nested in German planning regions. *Dependent variable is perceived importance of climate change measured on a five-point scale ranging between 1 "totally unimportant" and 5 "very important".* All continuous variables were z-standardized beforehand.

Using fractional weights to aggregate opinions

In the main analysis, we modeled aggregated binary outcomes using generalized linear multilevel models. Hence, we regressed a binary outcome on multiple predictor variables. To get a binary outcome variable that quantifies aggregated climate change opinions of individuals, we coded respondents who belief that climate change is already taking place, are concerned, and perceive the fight against climate change as important with 1 and 0 otherwise. To test the effect of the strict coding scheme on the regression results, we re-estimated the models on aggregate opinions across the three dimensions without transforming the ordinal responses to binary ones. Response scales, however, vary between the dimensions. Climate change belief is measured on a four-point, concern on a seven-point, and perceived importance on a five-point response scale. Simply summing up the responses across the varying scales to construct an aggregated opinion score is not possible without prioritizing one dimension over the other. Responses regarding concern (seven-point scale), would weight more than respondents' belief in climate change (four-point scale). To avoid this as best as possible, we harmonized the different scales by using fractional weights. Specifically, we multiplied respondents' belief with 1/4, their concern with 1/7, and their perceived importance with 1/5. The final score ranges between zero and three. We used the score as an alternative dependent variable and regressed it on the set of predictors using linear multilevel regressions. The estimated results are very similar to those obtained using a binary response scale. Hence, the recoding did not affect our results substantially.

Supplementary Table 8: *Regression results of linear multilevel model with weighted CCO score as dependent variable*

	**Without local context**	**Urban vs. Rural**	**Prospering vs. Declining**	**Green vs. Non-green**	**Green individual vs. Green regional**	**East vs. West**
	β (P) [95% CI]	*β* (P) [95% CI]	*β* (P) [95% CI]	*β* (P) [95% CI]	*β* (P) [95% CI]	*β* (P) [95% CI]
Household level						
(Intercept)	2.51 (<0.001) [2.48; 2.54]	2.51 (<0.001) [2.48; 2.54]	2.51 (<0.001) [2.48; 2.54]	2.50 (<0.001) [2.47; 2.54]	2.50 (<0.001) [2.47; 2.53]	2.54 (<0.001) [2.51; 2.57]
Sex (1=Male)	-0.11 (<0.001) [-0.13; -0.08]	-0.11 (<0.001) [-0.13; -0.08]	-0.11 (<0.001) [-0.13; -0.08]	-0.11 (<0.001) [-0.13; -0.08]	-0.11 (<0.001) [-0.13; -0.08]	-0.11 (<0.001) [-0.13; -0.09]
Age	0.19 (<0.001) [0.11; 0.27]	0.19 (<0.001) [0.11; 0.27]	0.20 (<0.001) [0.12; 0.27]	0.20 (<0.001) [0.12; 0.28]	0.20 (<0.001) [0.12; 0.28]	0.19 (<0.001) [0.11; 0.27]
Age (Squared)	-0.17 (<0.001) [-0.24; -0.09]	-0.17 (<0.001) [-0.25; -0.09]	-0.17 (<0.001) [-0.25; -0.10]	-0.18 (<0.001) [-0.25; -0.10]	-0.18 (<0.001) [-0.25; -0.10]	-0.17 (<0.001) [-0.24; -0.09]
Education (1= High School)	0.01 (0.551) [-0.02; 0.03]	0.01 (0.599) [-0.02; 0.03]	0.01 (0.515) [-0.02; 0.03]	0.01 (0.609) [-0.02; 0.03]	0.01 (0.616) [-0.02; 0.03]	0.01 (0.296) [-0.01; 0.04]
Income	-0.01 (0.017) [-0.01; -0.00]	-0.01 (0.018) [-0.01; -0.00]	-0.01 (0.01) [-0.01; -0.00]	-0.01 (0.013) [-0.01; -0.00]	-0.01 (0.013) [-0.01; -0.00]	-0.01 (0.004) [-0.01; -0.00]
Political Affiliation (1=Green Party)	0.24 (<0.001) [0.20; 0.29]	0.24 (<0.001) [0.20; 0.29]	0.24 (<0.001) [0.20; 0.28]	0.24 (<0.001) [0.20; 0.28]	0.26 (<0.001) [0.21; 0.31]	0.24 (<0.001) [0.19; 0.28]
Environmental Concern	0.14 (<0.001) [0.11; 0.17]	0.14 (<0.001) [0.11; 0.17]	0.14 (<0.001) [0.10; 0.17]	0.14 (<0.001) [0.10; 0.17]	0.14 (<0.001) [0.10; 0.17]	0.13 (<0.001) [0.10; 0.17]
Regional level						
Urban		0.01 (0.267) [-0.01; 0.02]				
Prospering			0.03 (<0.001) [0.02; 0.05]			
Green				0.03 (<0.001) [0.02; 0.04]	0.03 (<0.001) [0.02; 0.05]	
East						-0.10 (<0.001) [-0.13; -0.07]
Cross-level interaction						
Political Affiliation x Green					-0.03 (0.149) [-0.08; 0.01]	
AIC	10,132.21	10,141.10	10,123.08	10,121.62	10,127.23	10,105.54
BIC	10,201.25	10,217.04	10,199.02	10,197.57	10,210.07	10,181.48
R^2 (conditional)	0.054	0.054	0.055	0.055	0.055	0.056
R^2 (marginal)	0.048	0.048	0.052	0.052	0.052	0.054
n individuals	7,361	7,361	7,361	7,361	7,361	7,361
n regions	96	96	96	96	96	96
Variance (regions)	0.00	0.00	0.00	0.00	0.00	0.00

Linear multilevel models with households nested in German planning regions. *Dependent variable (CCO Weighted Sum) equals the sum of households' responses across the three items belief, concern, and perceived importance. Note that each item was measured on a different scale. To sum up the responses across the three items, we weighted households' responses for each item given the original scale. That is, we weighted households' belief with 1/4, their concern with 1/7, and their perceived importance with 1/5 such that respondents could gain a total sum of 3.* All continuous variables were z-standardized beforehand.

Supplementary Note 7

Green vote shares for additional general elections

In the main analysis, we indicated green regional cultures based on green vote shares from the German general election 1994. The primary reason to use regional green vote shares from 1994 was to reduce endogeneity concerns such that green vote share do not primarily represent the regional level of CCOs today, but a more general level of regional green culture manifested historically. To analyze to what extent our results depend on the general election of 1994, we computed additional multilevel models in which we extend green election results to all six general elections between 1994 and 2013. The results are reported in Supplementary Table 9. We first used the green vote shares from each election in separate models. Pairwise correlations range between 0.93 (1994 and 2009) and 0.98 (2009 and 2013). Green vote shares are therefore very stable over time. It is thus very unlikely that our results are driven by the 1994 election results only. Second, we also used the average green vote share across all elections as a predictor. In sum, the estimated coefficients range between 1.09 and 1.13 and the corresponding confidence intervals take values between 1.03 and 1.20. The results are therefore extremely robust across all specifications. Green vote shares from 1994, as used in the main text, are therefore a valid indication of green cultures and do not drive our results.

Supplementary Table 9: *Regression results for green vote shares from German general elections 1994-2013 as main independent variable*

	**Green 94**	**Green 98**	**Green 02**	**Green 05**	**Green 09**	**Green 13**	**Green 94-13**
	exp(*β*) (P) [95% CI]	exp(*β*) (P) [95% CI]	exp(*β*) (P) [95% CI]	exp(*β*) (P) [95% CI]	exp(*β*) (P) [95% CI]	exp(*β*) (P) [95% CI]	exp(*β*) (P) [95% CI]
Household level							
(Intercept)	1.06 (0.363)	1.07 (0.331)	1.07 (0.352)	1.07 (0.324)	1.07 (0.326))	1.07 (0.331)	1.07 (0.345)
	[0.93; 1.22]	[0.93; 1.22]	[0.93; 1.22]	[0.94; 1.22]	[0.94; 1.22]	[0.93; 1.22]	[0.93; 1.22]
Sex	0.83 (<0.001)	0.83 (<0.001)	0.83 (<0.001)	0.83 (<0.001)	0.83 (<0.001)	0.83 (<0.001)	0.83 (<0.001)
(1=Male)	[0.75; 0.92]	[0.75; 0.92]	[0.75; 0.92]	[0.75; 0.92]	[0.75; 0.92]	[0.75; 0.92]	[0.75; 0.92]
Age	1.63 (0.004)	1.62 (0.005)	1.63 (0.004)	1.63 (0.004)	1.64 (0.004)	1.64 (0.004)	1.63 (0.004)
	[1.17; 2.28]	[1.16; 2.26]	[1.17; 2.27]	[1.16; 2.27]	[1.17; 2.29]	[1.18; 2.29]	[1.17; 2.28]
Age (Squared)	0.60 (0.003)	0.61 (0.003)	0.60 (0.003)	0.60 (0.003)	0.60 (0.003)	0.60 (0.003)	0.60 (0.003)
	[0.43; 0.84]	[0.44; 0.85]	[0.43; 0.84]	[0.43; 0.84]	[0.43; 0.84]	[0.43; 0.84]	[0.43; 0.84]
Education	1.13 (0.034)	1.13 (0.035)	1.13 (0.035	1.13 (0.034)	1.13 (0.031)	1.13 (0.031)	1.13 (0.033)
(1= High School)	[1.01; 1.26]	[1.01; 1.26]	[1.01; 1.26]	[1.01; 1.26]	[1.01; 1.27]	[1.01; 1.27]	[1.01; 1.26]
Income	0.99 (0.321)	0.99 (0.342)	0.99 (0.329)	0.99 (0.337)	0.99 (0.316)	0.99 (0.303)	0.99 (0.323)
	[0.97; 1.01]	[0.97; 1.01]	[0.97; 1.01]	[0.97; 1.01]	[0.97; 1.01]	[0.97; 1.01]	[0.97; 1.01]
Political Affiliation	3.63 (<0.001)	3.51 (<0.001)	3.52 (<0.001)	3.53 (<0.001)	3.50 (<0.001)	3.53 (<0.001)	3.53 (<0.001)
(1=Green Party)	[2.76; 4.79]	[2.67; 4.61]	[2.67; 4.63]	[2.69; 4.63]	[2.67; 4.58]	[2.69; 4.64]	[2.68; 4.64]
Environmental	1.87 (<0.001)	1.88 (<0.001)	1.88 (<0.001)	1.88 (<0.001)	1.87 (<0.001)	1.87 (<0.001)	1.87 (<0.001)
	[1.62; 2.17]	[1.62; 2.18]	[1.62; 2.18]	[1.62; 2.18]	[1.62; 2.17]	[1.61; 2.17]	[1.62; 2.17]
Regional level							
Green	1.12 (<0.001)	1.09 (0.002)	1.10 (<0.001)	1.09 (0.002)	1.12 (<0.001)	1.13 (<0.001)	1.11 (<0.001)
	[1.06; 1.18]	[1.03; 1.16]	[1.04; 1.16]	[1.03; 1.15]	[1.05; 1.18]	[1.07; 1.20]	[1.05; 1.18]
Cross-level interaction							
Political Affiliation x	0.95 (0.674)	1.01 (0.930))	1.01 (0.960)	1.00 (0.982)	1.01 (0.926)	0.99 (0.935)	1.00 (0.980)
Green	[0.75; 1.21]	[0.81; 1.26]	[0.81; 1.25]	[0.81; 1.25]	[0.80; 1.28]	[0.77; 1.27]	[0.79; 1.26]
AIC	9,883.10	9,888.32	9,886.17	9,888.01	9,883.27	9,880.48	9,884.56
BIC	9,959.04	9,964.27	9,962.11	9,963.95	9,959.21	9,956.42	9,960.50
R^2 (conditional)	0.066	0.067	0.067	0.066	0.067	0.067	0.067
R^2 (marginal)	0.062	0.061	0.062	0.061	0.062	0.063	0.062
n individuals	7,361	7,361	7,361	7,361	7,361	7,361	7,361
n regions	96	96	96	96	96	96	96
Variance (regions)	0.01	0.02	0.02	0.02	0.02	0.01	0.02

Generalized linear multilevel models with households nested in German planning regions. *Dependent variable (CCO) equals 1 if household believes in climate change, is concerned, and perceives collective responses as important; and 0 otherwise. 53% were coded as 1 and 47% as 0*. Coefficients represent odd ratios. All continuous variables were z-standardized beforehand.

Supplementary Note 8

Controlling for East-West differences

The differences between East and West as reported in our main analysis are substantial. East and West Germany also differ in many other geographic features including economic prosperity, urban structure, and voting behavior. The East-West might therefore be a potential confounder. That is why we re-estimated the models for the three contextual variables *urban*, *prospering*, and *green* but this time we included the *East* dummy in each model to control for the general differences between East and West (see Models 1-3 in Supplementary Table 10). Although the point estimates change slightly, the main results remain unchanged. Hence, the general differences between East and West do not affect our regression results from the main text.

Supplementary Table 10: *Regression results with East-West dummy as control variable and selective migration*

	**Urban vs. Rural**	**Prospering vs. Declining**	**Green vs. Non-Green**	**Selective Migration**
	exp(*β*) (P) [95% CI]	exp(*β*) (P) [95% CI]	exp(*β*) (P) [95% CI]	exp(*β*) (P) [95% CI]
**Household level**				
(Intercept)	1.18 (0.021)	1.18 (0.017)	1.16 (0.032)	1.21 (0.009)
	[1.03; 1.35]	[1.03; 1.35]	[1.01; 1.34]	[1.05; 1.39]
Sex(1=Male)	0.82 (<0.001)	0.82 (<0.001)	0.82 (<0.001)	0.81 (<0.001)
	[0.74; 0.91]	[0.74; 0.91]	[0.74; 0.91]	[0.74; 0.90]
Age	1.59 (0.006)	1.63 (0.004)	1.62 (0.004)	1.58 (0.007)
	[1.14; 2.22]	[1.17; 2.27]	[1.16; 2.26]	[1.14; 2.21]
Age (Squared)	0.62 (0.004)	0.61 (0.003)	0.61 (0.003)	0.62 (0.005)
	[0.44; 0.86]	[0.43; 0.84]	[0.43; 0.84]	[0.45; 0.87]
Education	1.15 (0.015)	1.16 (0.010)	1.15 (0.013)	1.16 (0.010)
	[1.03; 1.29]	[1.04; 1.30]	[1.03; 1.29]	[1.04; 1.30]
Income	0.99 (0.201)	0.99 (0.142)	0.99 (0.174)	0.99 (0.180)
	[0.97; 1.01]	[0.97; 1.00]	[0.97; 1.01]	[0.97; 1.01]
Political Affiliation	3.51 (<0.001)	3.50 (<0.001)	3.49 (<0.001)	3.52 (<0.001)
(1=Green Party)	[2.78; 4.43]	[2.77; 4.41]	[2.77; 4.41]	[2.79; 4.45]
Environmental Concern	1.86 (<0.001)	1.85 (<0.001)	1.86 (<0.001)	1.86 (<0.001)
	[1.61; 2.16]	[1.60; 2.14]	[1.60; 2.15]	[1.60; 2.15]
**Regional level**				
Urban	1.05 (0.041)			
	[1.00; 1.10]			
Prospering		1.08 (0.003)		
		[1.03; 1.14]		
Green			1.07 (0.015)	
			[1.01; 1.12]	
East	0.72 (<0.001)	0.77 (<0.001)	0.77 (<0.001)	0.68 (0.022)
	[0.63; 0.82]	[0.68; 0.87]	[0.67; 0.88]	[0.49; 0.95]
Migration Ratio				1.00 (0.943)
				[0.91; 1.09]
**Regional level interaction**				
East x Migration Ratio				0.97 (0.825)
				[0.75; 1.25]
AIC	9871.42	9868.12	9870.14	9877.12
BIC	9947.36	9944.06	9946.08	9959.97
R^2 (conditional)	0.065	0.064	0.065	0.066
R^2 (marginal)	0.064	0.064	0.064	0.064
N individuals	7,361	7,361	7,361	7,361
N regions	96	96	96	96
Variance (regions)	0.00	0.00	0.00	0.01

Binary logistic multilevel models with households nested in German planning regions. *Dependent variable (CCO) equals 1 if household believes in climate change, is concerned, and perceives collective responses as important; and 0 otherwise.* Coefficients represent odd ratios. 95% confidence intervals in square brackets. All continuous variables were z-standardized beforehand.

Supplementary Note 9

Selective migration

As reported in previous research[25], regions in East Germany suffered severely from out-migration, particularly in the first few years after reunification. Specifically, younger and better educated people left East Germany in search for a more promising future in West Germany.[26] Specific socio-demographic characteristics such as age, gender, and education are associated with climate change opinions.[27] The observed differences in public CCOs between East and West might therefore be more the result of selective migration of specific demographic groups shortly after reunification than an outcome of the two fundamentally different political and ideological systems.

To test the influence of selective migration, we collected data from the *Federal Statistical Office Germany (DESTATIS)* (2021) on domestic German migration between 1991 and 1992 at the district level. We re-aggregated the data from the district level to the level of the 96 planning regions used in the regression analysis based on the official municipality key. Based on this migration data, we calculated the net-migration for the 96 planning regions in Germany shortly after reunification.

We included the net-migration rate into our regression analysis alongside the East-West dummy (see Model 4 in Supplementary Table 10). Although regions in the East (Mean = 0.767, CI = [0.72; 0.814]) compared to those in the West (Mean = 1.09, CI = [1.06; 1.12]) experienced higher out-migration than in-migration (net migration ratio < *1*), the net migration ratio in general is not associated with individual CCOs (exp(β) = 1.00, p = 0.943). Furthermore, we included an interaction term of the net migration ratio and the East-West dummy to examine whether individuals residing in East German regions, which experienced high rates of out-migration in the early 1990s, have lower CCOs today compared to people residing in East German regions with lower out-migration. The interaction term, however, is statistically not significant (exp(β) = 0.97, p = 0.825). That is, people residing in East German regions that lost a substantial part of their population due to migration, do not have significantly different CCOs than individuals residing in East Germany but in regions with lower historical rates of out-migration.

We also examined the potential effect of selective migration in East German regions on today's CCOs at the municipality level and divided the sample of Eastern municipalities into quartiles based on the net migration ratio observed at the district level. Note that historical migration statistics are not available at the municipality level. Group 1 summarizes municipalities located in districts that experienced the largest out-migration (Mean = 0.604, CI = [0.561; 0.647]) and group 4 summarizes municipalities with the lowest out-migration (Mean = 0.973, CI = [0.927; 1.02]). Based on this grouping, we calculated the mean of every CCO dimension for each of the four groups. Supplementary Figure 8 depicts the results. If selective migration played a substantial role for the geographic dispersion of today's CCOs, we expect group 1 to have significant lower CCOs than municipalities belonging to group 4. Except from the belief in climate change (panel A, Supplementary Figure 8), we observe significant lower opinions of Eastern municipalities that experienced more severe out-migration. Yet, even the municipalities with a net migration ratio of around *1* (group 4), show substantial negative deviations from the national average.

We repeated the same analysis for West German municipalities and Supplementary Figure 9 reports the results. We find a similar pattern for the sample of West German municipalities: Municipalities with higher out-migration values tend to have lower CCOs, which corresponds to our findings that

there are substantial differences between prospering and declining regions. Taken together, our analysis suggests that selective domestic migration has played a role in shaping the geography of today's CCOs for both parts of the country. It is, therefore, very unlikely that selective migration represents the primary cause for the observed differences between East and West German municipalities in today's CCOs.

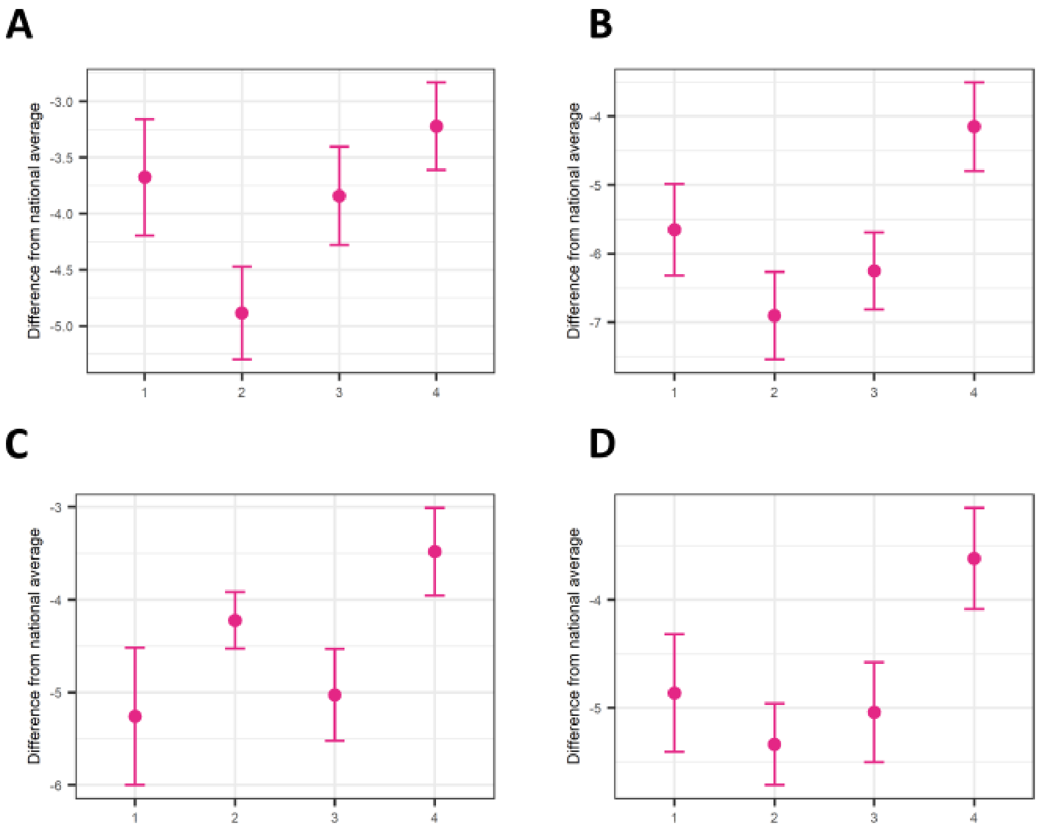

Supplementary Figure 8: *Average deviation from the national average in East German municipalities for* ***(A)*** *belief,* ***(B)*** *concern,* ***(C)*** *importance, and* ***(D)*** *the overall difference from the national average. The sample of East German municipalities (N = 927) was divided into quartiles based on the net migration ratio in 1991 and 1992 at the corresponding district level. Average net migration ratios of the four groups: Group 1 = 0.604, Group 2 = 0.723, Group 3 = 0.8, and Group 4 = 0.973. Error bars represent 95% confidence intervals of SEM.*

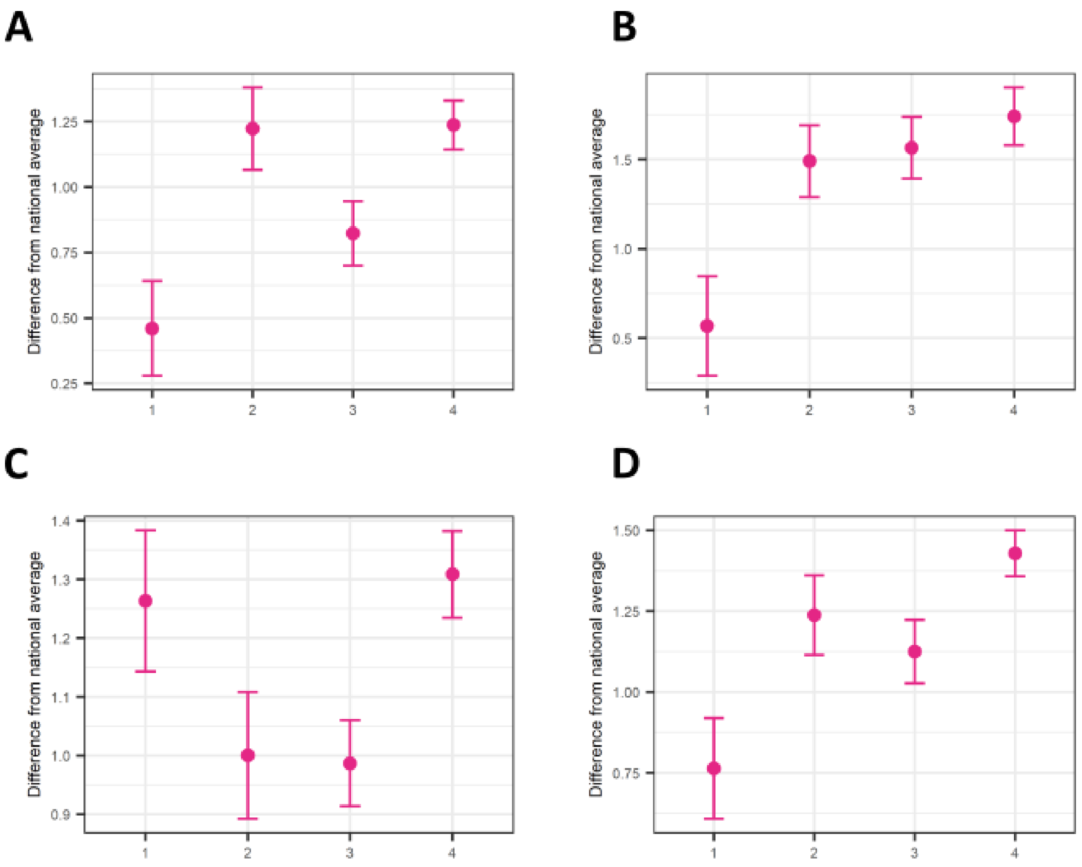

Supplementary Figure 9: *Average deviation from the national average in West German municipalities for* ***(A)*** *belief,* ***(B)*** *concern,* ***(C)*** *importance, and* ***(D)*** *the overall difference from the national average. The sample of West German municipalities (N = 3,644) was divided into quartiles based on the net migration ratio in 1991 and 1992 at the corresponding district level. Average net migration ratios of the four groups: Group 1 = 0.943, Group 2 = 1.08, Group 3 = 1.15, and Group 4 = 1.26. Error bars represent 95% confidence intervals of SEM.*

Supplementary Note 10

Composition of regional samples after applying actor-based clustering

As previous research showed that individual CCOs depend on socio-demographic characteristics such as gender or age[27] sampling bias is an important issue when locating households to their regions and mapping regional CCOs. The advantage of our actor-based clustering approach is that the complete sample of households is included in the calculation of local CCOs by assigning more weights to proximate and less weights to distant households. Due to the geographic weighting, however, local samples can be biased if they include, for example, only male respondents or older household leaders in close geographic proximity. Although this is highly unlikely, as the spatial smoothing function includes the complete household survey, we nevertheless calculated the average age and the share of male household leaders within a distance of $<= r$ with $r = 60km$, as the distance of *60km* represents the distance when geographic weights decrease to *0.5* (see Methods in the main text).

In the complete sample, the heads of the surveyed households were on average 53 years old. The average age in municipalities ranges between 47 and 58 years. 85% percent of the observations range between an average age of 50 to 55 years. Comparing the range to official statistics at the level of municipalities is not possible due to data availability. Hence, we compare the range of average age in our sample of municipalities (N = 4,677) to the range at the district level (N = 401) as reported in data collected from the *Federal Institute for Research on Building, Urban Affairs and Spatial Development (BBSR).* The average age across the 401 counties ranges between 40 and 50, which is a total range of 11 years. When comparing the ranges, it is important to consider that the household survey only reports the age of the heads of the surveyed households and only includes persons older than 18 years whereas official statistics include all people living in a region resulting in an overestimation of the average age in our sample. Hence, the total range of 11 years (household survey) and 10 years (range in official statistics) suggests that the regional samples are not severely biased towards younger or older people.

Regarding gender, 65% of the heads of the surveyed households was male. Males are typically over-represented in household surveys. The gender of the survey respondents is therefore representative at the household level. The local shares range between 47% and 86% males. 72% of the local values range between 60% and 70% male household leaders. Hence, we cannot detect a severe bias in terms of gender. It is not possible to compare these numbers to official statistics; as public statistics only provide the share of males in the general population.

Supplementary Note 11

Different specification of the spatial smoothing function – maps and correlations

We produced the maps, as presented in the main text, based on a spatial smoothing function that applies actor-based clustering[21]. To generate the maps, we needed to determine two unknown parameters *r* (the distance in kilometers when spatial weights decrease to exactly 0.5) and s (the slope of the log-logistic distance decay function defining how fast spatial weights decrease). Basically, these two parameters determine the spatial reach of the spatial smoothing. Higher values of *r* and lower values of *s* increase the spatial smoothing. In the main text, we defined *r* to be *60* kilometers and *s* to be *7* based on a trade-off between the reasonable reach of spatial interactions of people indicated by commuting patterns [22] and the density of information necessary to provide accurate estimates (see section on cross-validation). To verify our results as reported in the main text, we repeated the geographic analysis and estimated the local values based on *r* equaling *50* and *70* kilometers while holding *s* constant at *7* (Supplementary Figures 10 and 11) and *s* to be *6* and *8* while holding *r* constant at *60* (Supplementary Figures 12 and 13). The estimated local values are very similar for all specification of *r* and *s*. Supplementary Tables 11-13 report the correlations of the local estimates based on the different definitions of *r* and *s* for all three CCO dimensions. Correlations range between 0.938 and 0.999 (belief), 0.952 and 0.999 (concern), and 0.955 and 0.999 (importance) (see Supplementary Tables 11-13).

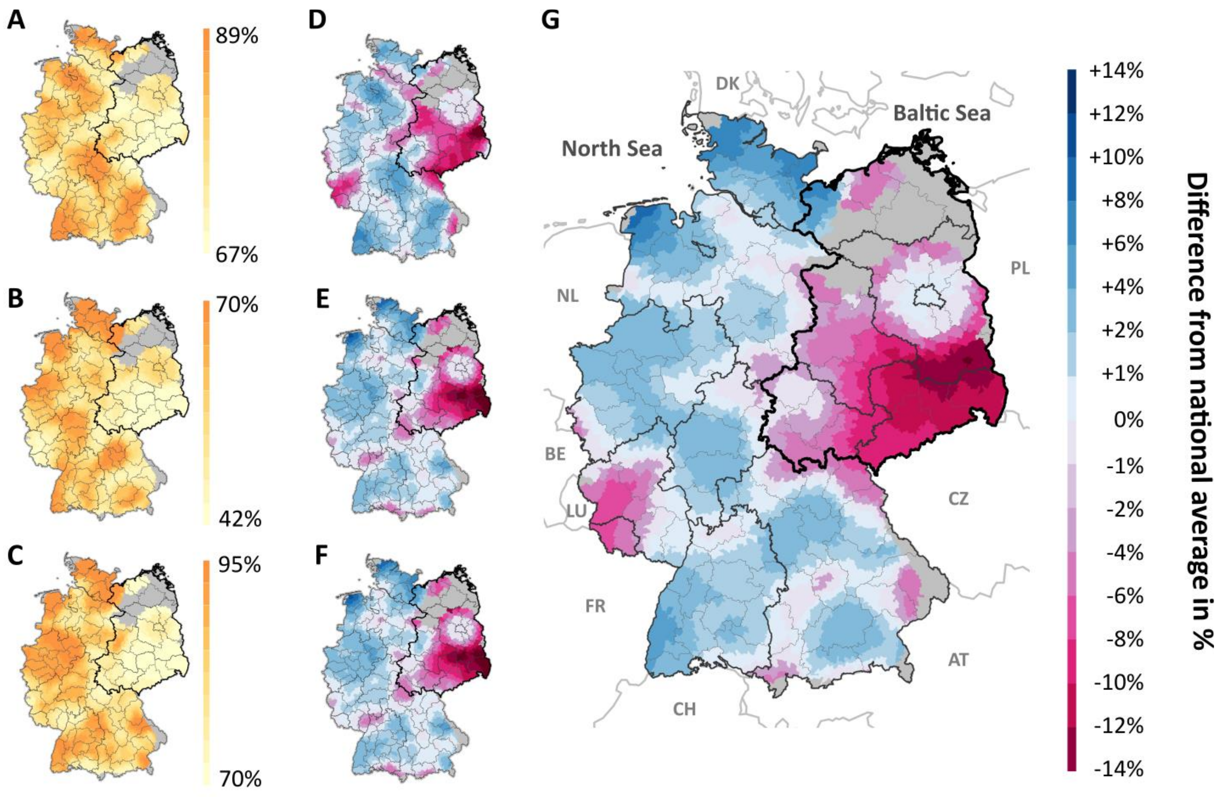

Supplementary Figure 10: *Geographic distribution of public CCOs across German municipalities with r = 50km and s = 7. The shares of local populations that **(A)** believe in climate change, **(B)** are concerned, and **(C)** perceive collective responses to be important. The percentage difference from the national average is visualized in panel **(D)** for belief, **(E)** concern, **(F)** importance. The average difference from the national average across the three dimensions reported in (**D–F)** is depicted in **(G)**. The bold black line indicates the former division into East and West Germany. Solid black lines indicate the 16 federal states. Solid grey lines indicate the 96 planning regions used for the multilevel estimations. Municipalities with too little information are colored in grey.*

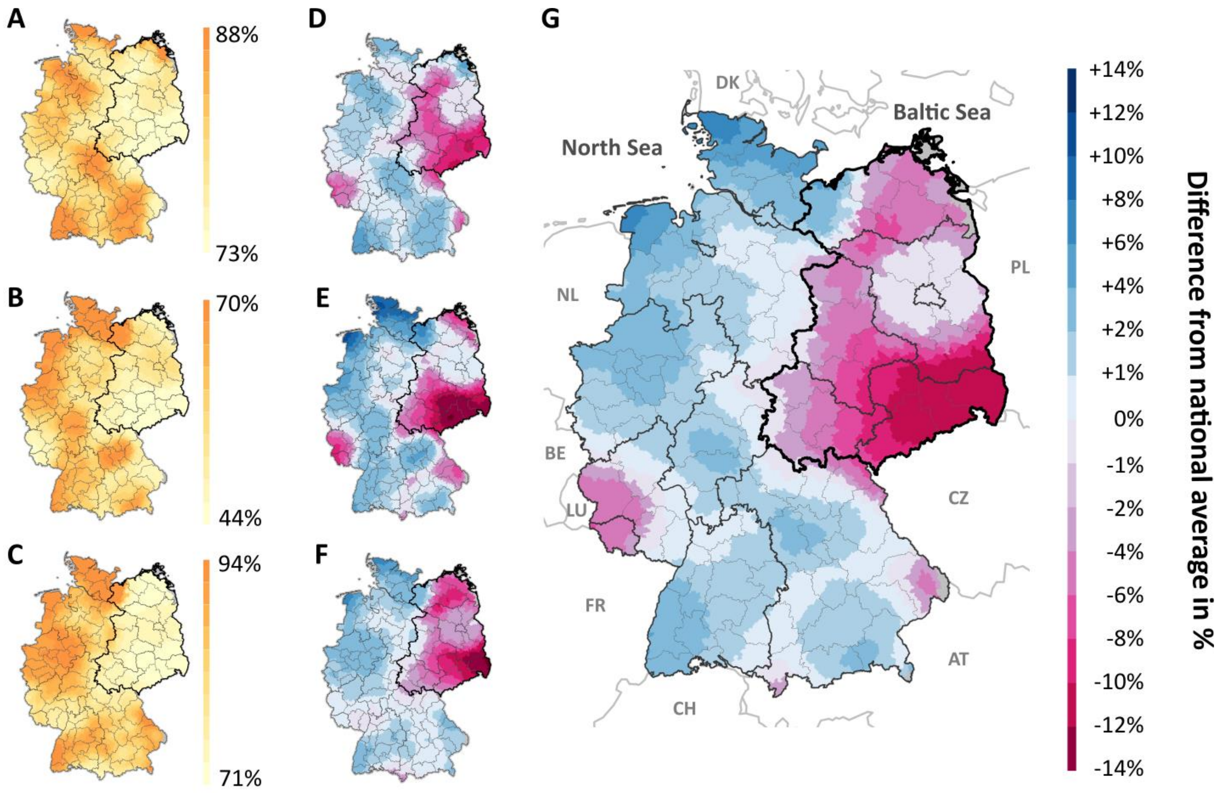

Supplementary Figure 11: *Geographic distribution of public CCOs across German municipalities with r = 70km and s = 7. The shares of local populations that* ***(A)*** *believe in climate change,* ***(B)*** *are concerned, and* ***(C)*** *perceive collective responses to be important. The percentage difference from the national average is visualized in panel* ***(D)*** *for belief,* ***(E)*** *concern,* ***(F)*** *importance. The average difference from the national average across the three dimensions reported in (****D–F)*** *is depicted in* ***(G)****. The bold black line indicates the former division into East and West Germany. Solid black lines indicate the 16 federal states. Solid grey lines indicate the 96 planning regions used for the multilevel estimations. Municipalities with too little information are colored in grey.*

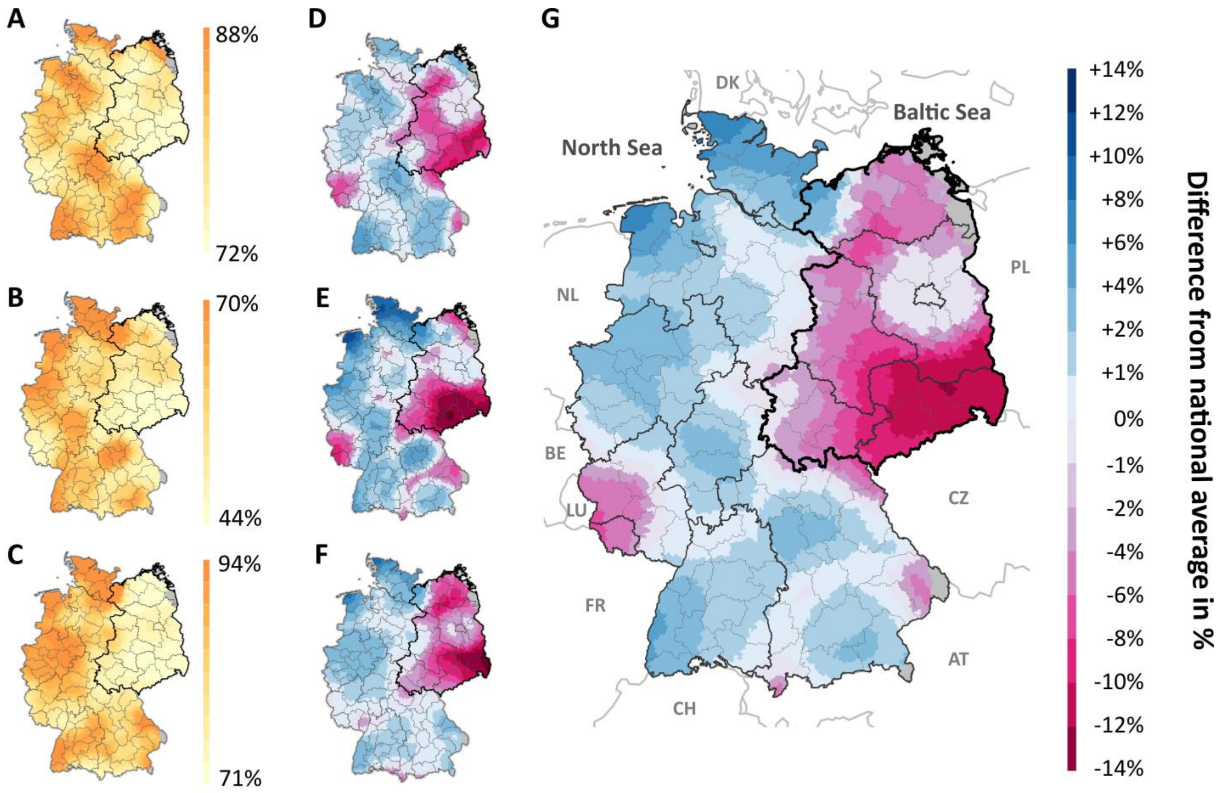

Supplementary Figure 12: *Geographic distribution of public CCOs across German municipalities with r = 60km and s = 6. The shares of local populations that* ***(A)*** *believe in climate change,* ***(B)*** *are concerned, and* ***(C)*** *perceive collective responses to be important. The percentage difference from the national average is visualized in panel* ***(D)*** *for belief,* ***(E)*** *concern,* ***(F)*** *importance. The average difference from the national average across the three dimensions reported in (****D–F)*** *is depicted in* ***(G)****. The bold black line indicates the former division into East and West Germany. Solid black lines indicate the 16 federal states. Solid grey lines indicate the 96 planning regions used for the multilevel estimations. Municipalities with too little information are colored in grey.*

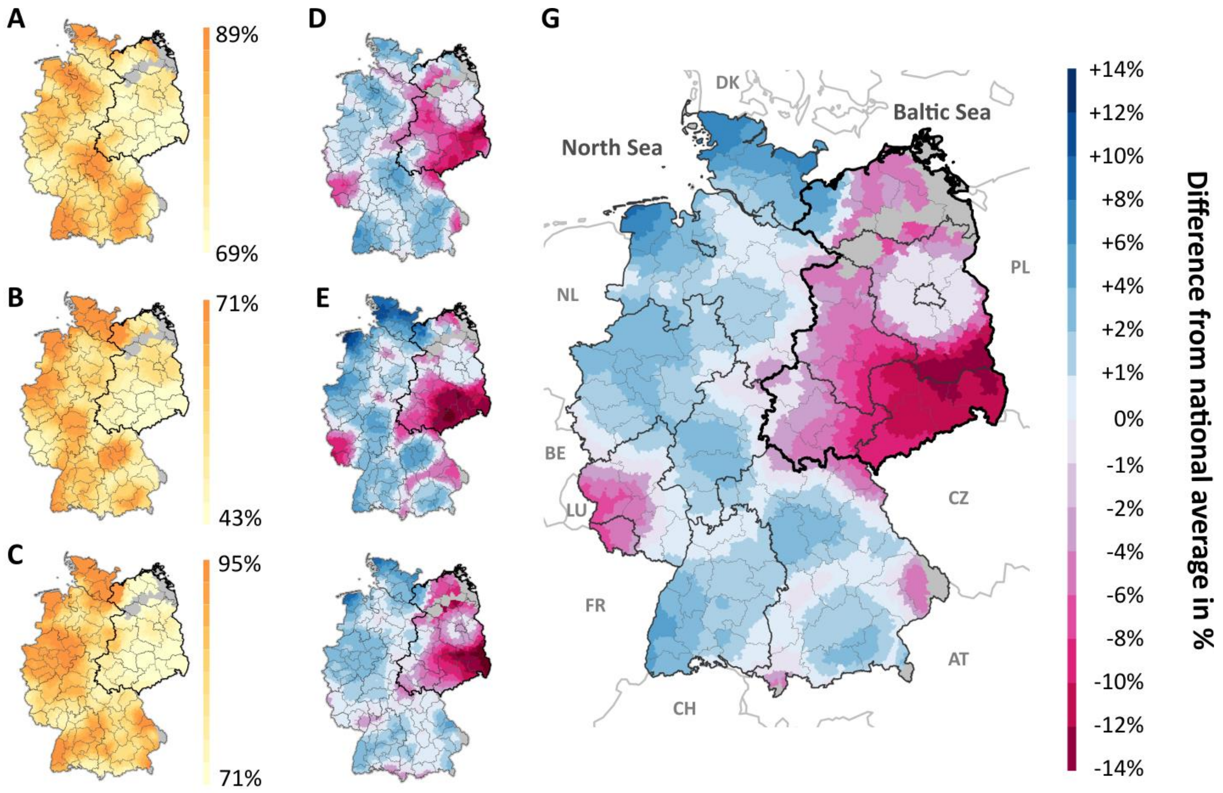

Supplementary Figure 13: *Geographic distribution of public CCOs across German municipalities with r = 60km and s = 8. The shares of local populations that* ***(A)*** *believe in climate change,* ***(B)*** *are concerned, and* ***(C)*** *perceive collective responses to be important. The percentage difference from the national average is visualized in panel* ***(D)*** *for belief,* ***(E)*** *concern,* ***(F)*** *importance. The average difference from the national average across the three dimensions reported in (****D–F)*** *is depicted in* ***(G)****. The bold black line indicates the former division into East and West Germany. Solid black lines indicate the 16 federal states. Solid grey lines indicate the 96 planning regions used for the multilevel estimations. Municipalities with too little information are colored in grey.*

Supplementary Table 11: *Correlation table of CCO dimension belief for different specification of r and s applied in the spatial smoothing function*

	r = 50km, s = 7	***r = 60km, s = 7***	***r = 70km, s = 7***	***r = 60km, s = 6***	***r = 60km, s = 8***
r = 50km, s = 7	1				
r = 60km, s = 7	0.980	1			
r = 70km, s = 7	0.938	0.987	1		
r = 60km, s = 6	0.974	0.999	0.992	1	
r = 60km, s = 8	0.983	0.999	0.981	0.997	1

Supplementary Table 12: *Correlation table of CCO dimension concern for different specification of r and s applied in the spatial smoothing function*

	r = 50km, s = 7	***r = 60km, s = 7***	***r = 70km, s = 7***	***r = 60km, s = 6***	***r = 60km, s = 8***
r = 50km, s = 7	1				
r = 60km, s = 7	0.984	1			
r = 70km, s = 7	0.952	0.990	1		
r = 60km, s = 6	0.980	0.999	0.994	1	
r = 60km, s = 8	0.986	0.999	0.986	0.997	1

Supplementary Table 13: *Correlation table of CCO dimension importance for different specification of r and s applied in the spatial smoothing function*

	r = 50km, s = 7	***r = 60km, s = 7***	***r = 70km, s = 7***	***r = 60km, s = 6***	***r = 60km, s = 8***
r = 50km, s = 7	1				
r = 60km, s = 7	0.984	1			
r = 70km, s = 7	0.955	0.991	1		
r = 60km, s = 6	0.980	0.999	0.995	1	
r = 60km, s = 8	0.987	0.999	0.987	0.997	1

Supplementary Note 12

Excluding Berlin

Berlin represents an outlier with its unique history as a divided city during the German separation between 1949 and 1990. Since Berlin represents the largest sample size, Berlin could therefore affect the regression analysis substantially. To test whether Berlin drives our results, we repeated the empirical analysis from the main text and excluded Berlin from the estimations. As reported in Supplementary Table 14, the main results remain unchanged. Excluding Berlin form the analysis, however, results in more pronounced East-West differences. The East dummy decreased from 0.71 in the main regression analysis to 0.67 in the regression without Berlin.

Supplementary Table 14: *Regression results without Berlin*

	**Urban vs. Rural**	**Prospering vs. Declining**	**Green vs. Non-green**	**East vs. West**
	exp(β) (P) [95% CI]	exp(β) (P) [95% CI]	exp(β) (P) [95% CI]	exp(β) (P) [95% CI]
Household level				
(Intercept)	1.07 (0.372)	1.09 (0.213)	1.07 (0.352)	1.20 (0.011)
	[0.93; 1.23]	[0.95; 1.25]	[0.93; 1.22]	[1.04; 1.38]
Sex (1=Male)	0.83 (<0.001)	0.83 (<0.001)	0.83 (<0.001)	0.82 (<0.001)
	[0.74; 0.92]	[0.74; 0.92]	[0.75; 0.92]	[0.73; 0.91]
Age	1.87 (<0.001)	1.92 (<0.001)	1.93 (<0.001)	1.89 (<0.001)
	[1.32; 2.64]	[1.36; 2.72]	[1.37; 2.73]	[1.34; 2.66]
Age (Squared)	0.54 (<0.001)	0.53 (<0.001)	0.52 (<0.001)	0.54 (<0.001)
	[0.38; 0.76]	[0.37; 0.74]	[0.37; 0.74]	[0.38; 0.75]
Education	1.14 (0.027)	1.16 (0.013)	1.15 (0.019)	1.18 (0.006)
	[1.02; 1.28]	[1.03; 1.30]	[1.02; 1.29]	[1.05; 1.32]
Income	0.99 (0.452)	0.99 (0.274)	0.99 (0.323)	0.99 (0.222)
	[0.97; 1.01]	[0.97; 1.01]	[0.97; 1.01]	[0.97; 1.01]
Political Affiliation	3.54 (<0.001)	3.51 (<0.001)	3.49 (<0.001)	3.47 (<0.001)
(1=Green Party)	[2.78; 4.51]	[2.75; 4.46]	[2.74; 4.44]	[2.72; 4.42]
Environmental Concern	1.89 (<0.001)	1.87 (<0.001)	1.87 (<0.001)	1.85 (<0.001)
	[1.63; 2.20]	[1.60; 2.17]	[1.61; 2.18]	[1.59; 2.16]
Regional level				
Urban	1.06 (0.046)			
	[1.00; 1.12]			
Prospering		1.14 (<0.001)		
		[1.07; 1.20]		
Green				0.67 (<0.001)
				[0.58; 0.78]
East			1.13 (<0.001)	
			[1.07; 1.19]	
AIC	9,308.94	9,296.42	9,296.73	9,288.00
BIC	9,377.38	9,364.85	9,365.16	9,356.44
R^2 (conditional)	0.066	0.066	0.067	0.066
R^2 (marginal)	0.060	0.063	0.063	0.065
N individuals	6,930	6,930	6,930	6,930
N regions	95	95	95	95
Variance (regions)	0.02	0.01	0.01	0.00

Binary logistic multilevel models with households nested in German planning regions. *Dependent variable (CCO) equals 1 if household believes in climate change, is concerned, and perceives collective responses as important; and 0 otherwise.* Coefficients represent odd ratios. 95% confidence intervals in square brackets. All continuous variables were z-standardized beforehand.

References

1. Frondel, M. *et al.* Socio-Ecological Panel, 1st Survey Wave. Sozioökologisches Panel, 1. Befragungswelle. 2,19 MB (2016) doi:10.7807/GREENSOEP:DE:V1.
2. Frondel, M. *et al.* Socio-Ecological Panel, 2nd Survey Wave. Sozio-Ökologisches Panel, 2. Befragungswelle. 1,75 MB (2016) doi:10.7807/GREENSOEP:DE:V2.
3. Frondel, M. *et al.* Socio-Ecological Panel, 3rd Survey Wave. Sozio-Ökologisches Panel, 3. Befragungswelle. (2016) doi:10.7807/GREENSOEP:DE:V3.
4. Frondel, M. *et al.* Socio-Ecological Panel, 4rd Survey Wave. Sozial-Ökologisches Panel, 4. Befragungswelle. 1.19 MB (2020) doi:10.7807/GREENSOEP:EN:V4.
5. Kussel, G. & Larysch, T. *Sozial-Ökologisches Panel: Datenbeschreibung der Haushaltsbefragung*. (RWI, 2017).
6. Klick, L., Kussel, G. & Sommer, S. Green-SÖP: The Socio-ecological Panel Survey: 2012–2016. *Jahrbücher für Nationalökonomie und Statistik* **241**, 405–414 (2021).
7. Lippmann, W. *Public Opinion*. (Harcourt, Brace and Company, 1922).
8. Shwom, R. L. *et al.* Public Opinion on Climate Change. in *Climate Change and Society* (eds. Dunlap, R. E. & Brulle, R. J.) 269–299 (Oxford University Press, 2015). doi:10.1093/acprof:oso/9780199356102.003.0009.
9. Capstick, S., Whitmarsh, L., Poortinga, W., Pidgeon, N. & Upham, P. International trends in public perceptions of climate change over the past quarter century: International trends in public perceptions of climate change. *Wires Clim. Change* **6**, 35–61 (2015).
10. Poortinga, W., Spence, A., Whitmarsh, L., Capstick, S. & Pidgeon, N. F. Uncertain climate: An investigation into public scepticism about anthropogenic climate change. *Glob. Environ. Change* **21**, 1015–1024 (2011).
11. Egan, P. J. & Mullin, M. Climate Change: US Public Opinion. *Annu. Rev. Polit. Sci.* **20**, 209–227 (2017).

12. Howe, P. D., Mildenberger, M., Marlon, J. R. & Leiserowitz, A. Geographic variation in opinions on climate change at state and local scales in the USA. *Nat. Clim. Change* **5**, 596–603 (2015).
13. Mildenberger, M. *et al.* The Distribution of Climate Change Public Opinion in Canada. *PLoS ONE* **11**, e0159774 (2016).
14. Nisbet, M. C. & Myers, T. The Polls Trends: Twenty Years of Public Opinion about Global Warming. *Public Opin. Quart.* **71**, 444–470 (2007).
15. Drews, S. & van den Bergh, J. C. J. M. What explains public support for climate policies? A review of empirical and experimental studies. *Clim. Policy* **16**, 855–876 (2016).
16. Gallup. Gallup poll social series: environment. (2021).
17. Poortinga, W., Whitmarsh, L., Steg, L., Böhm, G. & Fisher, S. Climate change perceptions and their individual-level determinants: A cross-European analysis. *Glob. Environ. Change* **55**, 25–35 (2019).
18. ESS. European Social Survey Round 8 Data. Data file edition 2.2. *Research Data, Norway – Data Archive and Distributor of ESS Data for ESS ERIC* (2016).
19. Gärtner, L. & Schoen, H. Experiencing climate change: revisiting the role of local weather in affecting climate change awareness and related policy preferences. *Climatic Change* **167**, 31 (2021).
20. Pacheco, J. Using National Surveys to Measure Dynamic U.S. State Public Opinion: A Guideline for Scholars and an Application. *State Politics and Policy Quarterly* **11**, 415–439 (2011).
21. Brenner, T. Identification of clusters: An actor-based approach. *Working Papers on Innovation and Space* **2**, (2017).
22. Kloas, J. & Kuhfeld, H. Entfernungspauschale: Bezieher hoherEinkommen begünstigt: aktuelle Ergebnisse zum Verkehrsverhalten privater Haushalte. *DIW Wochenbericht* **70**, (2003).
23. Hanretty, C. An Introduction to Multilevel Regression and Post-Stratification for Estimating Constituency Opinion. *Polit. Stud. Rev.* **18**, 630–645 (2020).

24. Broniecki, P., Leemann, L. & Wüest, R. Improved Multilevel Regression with Poststratification through Machine Learning (autoMrP). *J. Polit.* **84**, 597-601 (2022).

25. Uhlig, H. Regional Labor Markets, Network Externalities and Migration: The Case of German Reunification. *Am. Econ. Rev.* **96**, 383–387 (2006).

26. Leibert, T. She leaves, he stays? Sex-selective migration in rural East Germany. *J. Rural Stud.* **43**, 267–279 (2016).

27. Hornsey, M. J., Harris, E. A., Bain, P. G. & Fielding, K. S. Meta-analyses of the determinants and outcomes of belief in climate change. *Nat. Clim. Change* **6**, 622–626 (2016).
