# Supplementary material for: Exploring the variances of climate change opinions in Germany at a fine-grained local scale
Source: Nat Commun. 2024 Feb 29;15:1867. doi: 10.1038/s41467-024-45930-8 (PMC10904852; doi:10.1038/s41467-024-45930-8)
Supplement: Supplementary file 2 — Reporting Summary [file 41467_2024_45930_MOESM2_ESM.pdf]

nature portfolio

Corresponding author(s): Lars Mewes
Last updated by author(s): Jul 10, 2023

Reporting Summary

Nature Portfolio wishes to improve the reproducibility of the work that we publish. This form provides structure for consistency and transparency in reporting. For further information on Nature Portfolio policies, see our Editorial Policies and the Editorial Policy Checklist.

Statistics

For all statistical analyses, confirm that the following items are present in the figure legend, table legend, main text, or Methods section.

n/a	Confirmed	
☐	☒	The exact sample size (*n*) for each experimental group/condition, given as a discrete number and unit of measurement
☐	☒	A statement on whether measurements were taken from distinct samples or whether the same sample was measured repeatedly
☐	☒	The statistical test(s) used AND whether they are one- or two-sided<br>*Only common tests should be described solely by name; describe more complex techniques in the Methods section.*
☐	☒	A description of all covariates tested
☐	☒	A description of any assumptions or corrections, such as tests of normality and adjustment for multiple comparisons
☐	☒	A full description of the statistical parameters including central tendency (e.g. means) or other basic estimates (e.g. regression coefficient) AND variation (e.g. standard deviation) or associated estimates of uncertainty (e.g. confidence intervals)
☐	☒	For null hypothesis testing, the test statistic (e.g. *F*, *t*, *r*) with confidence intervals, effect sizes, degrees of freedom and *P* value noted<br>*Give P values as exact values whenever suitable.*
☒	☐	For Bayesian analysis, information on the choice of priors and Markov chain Monte Carlo settings
☐	☒	For hierarchical and complex designs, identification of the appropriate level for tests and full reporting of outcomes
☒	☐	Estimates of effect sizes (e.g. Cohen's *d*, Pearson's *r*), indicating how they were calculated

Our web collection on statistics for biologists contains articles on many of the points above.

Software and code

Policy information about availability of computer code

Data collection	The raw data was provided by the Leibniz Institute for Economic Research (see Kussel & Larysch 2017 for a detailed description). Any processing of the data was conducted in R (version 4.0.3) involving data cleaning, matching surveyed households with geo-data, conducting the geographic as well as statistical analysis. Variables at the municipality and regional level were obtained from the Federal Institute for Research on Building, Urban Affairs, and Spatial Development (BBSR) that provides publicly available data at inkar.de for a variety of spatial units.
Data analysis	The complete empirical analysis was conducted in R (version 4.0.3). To conduct the geographic analysis, we relied on the spdep (version 1.2), rgdal (version 1.5), and geospehere (version 1.5) package. For multilevel modelling, we used the glmer() from the lm4 package (version 1.1).

For manuscripts utilizing custom algorithms or software that are central to the research but not yet described in published literature, software must be made available to editors and reviewers. We strongly encourage code deposition in a community repository (e.g. GitHub). See the Nature Portfolio guidelines for submitting code & software for further information.

Data

Policy information about availability of data

All manuscripts must include a data availability statement. This statement should provide the following information, where applicable:

- Accession codes, unique identifiers, or web links for publicly available datasets
- A description of any restrictions on data availability
- For clinical datasets or third party data, please ensure that the statement adheres to our policy

The code of the geographic (spatial smoothing function and geographic mapping) as well as statistical analysis (multilevel regressions) can be made available to the public upon acceptance. The household-data is propriety data of the Leibniz Institute for Economic Research (RWI). The data is generally accessible for research purposes upon request at RWI. We received access to the data via a biliteral contract that prevents us from sharing the data publicly. In addition, the availability of the household data is restricted to secure anonymity of the surveyed households. Hence, the household-data may not currently be shared publicly. The geo-data including the geographic shapefiles used in the geographic analysis, as well as all variables at the regional level (used in the statistical analysis) are publicly available.

Human research participants

Policy information about studies involving human research participants and Sex and Gender in Research.

Reporting on sex and gender	In our study, we used data on respondents' gender as an important predictor variable to accurately model respondents' climate change opinions. Unfortunately, the secondary does not distinguish between genders other than female and male. Previous research has provided ample evidence indicating that females show higher awareness of climate change than males. Respondents self-reported their gender during the survey. The regression tables in manuscript and the supplementary material show the gender differences in climate change opinions. In our main analysis, females show ~22% higher odds of being aware of climate change than male respondents. Hence, our results confirm the gender bias in climate change opinions.
Population characteristics	See below.
Recruitment	The surveys were conducted as part of the Omninet panel by forsa, a German market research company. Households were selected to achieve representativeness of the population over 14 years of age in Germany. Participation in the survey is completely voluntary. Upon completion of the questionnaire, respondents received a small bonus/voucher that could be utilized in any payback-system.
Ethics oversight	For the present study, no ethical approval was necessary.

Note that full information on the approval of the study protocol must also be provided in the manuscript.

Field-specific reporting

Please select the one below that is the best fit for your research. If you are not sure, read the appropriate sections before making your selection.

☐ Life sciences ☒ Behavioural & social sciences ☐ Ecological, evolutionary & environmental sciences

For a reference copy of the document with all sections, see nature.com/documents/nr-reporting-summary-flat.pdf

Behavioural & social sciences study design

All studies must disclose on these points even when the disclosure is negative.

Study description	The present study uses a representative household survey from Germany to reveal climate change opinions at a fine-grained local scale to investigate geographic differences in localities in one country. We test the hypothesis that individual opinions – besides individual-level factors – depend on the local context.
Research sample	The representative household sample was conducted by the Leibniz Institute for Economic Research (RWI) between 2012 and 2015 (including 4 waves). The research sample is composed of 12,612 households in Germany. The respondents were the heads of the surveyed households defined as the person who decides about financial decisions and at least 18 years old. We only considered the most recent answers of the respondents. For the multilevel analysis, we combined three items from the survey to measure opinions on climate change (see main study) and, therefore, we only considered households that answered all three answers reducing n to 7,361 for the statistical analysis.
Sampling strategy	The household data was collected by the RWI. The sampling strategy of the RWI aimed at achieving a representative sample of the German population over 14 years of age (see data collection for details).
Data collection	The household data used in our study was collected by the Leibniz Institute for Economic Research (RWI) between 2012 and 2015 (see Kussel & Larysch 2017 for a detailed description) as part of the Socio-Ecological Panel. The survey was part of a larger research

project, which aimed at evaluating the perception of climate mitigation and adaption policies in the German population. On average, the age of the surveyed heads of the households is 53.2, 66.7% were male, and the average income is 2,823.25 Euro. The majority of households participated online. Those without internet access received a programmable device to participate. After completely participating in the survey, respondents received vouchers that could be used in any payback-system.

Timing

The survey was conducted between 2012 and 2015. We only considered the most recent answers of the households to avoid including households twice during the empirical analyses (i.e., to avoid double counting).

Data exclusions

The complete household survey is composed of 12,612 surveyed households. To answer our research question we applied the following exclusion restrictions: (1) In order to conduct a geographic analysis, we required the residence of the surveyed households. Fortunately, for all of the surveyed households, the place of residence was given. That is, no household was excluded because of missing geographic information. (2) To assess fine-grained geographic differences in climate change opinions, we required information on households' perception regarding climate change. We used three items from the survey: i) belief (n = 11,903), ii) concern (n = 8,487), and iii) importance (n = 12,337). To produce the maps (Figure 1 in the main study), we used the samples given for every item to minimize exclusion. To conduct the multilevel analysis, however, we required households to answer all questions, limiting the sample to 7,361 households.

Non-participation

Considering that we received a complete data set after sampling, we have no information about how many participants dropped out / declined participation during data collection of the RWI.

Randomization

N/A

Reporting for specific materials, systems and methods

We require information from authors about some types of materials, experimental systems and methods used in many studies. Here, indicate whether each material, system or method listed is relevant to your study. If you are not sure if a list item applies to your research, read the appropriate section before selecting a response.

Materials & experimental systems

n/a	Involved in the study
☒	☐ Antibodies
☒	☐ Eukaryotic cell lines
☒	☐ Palaeontology and archaeology
☒	☐ Animals and other organisms
☒	☐ Clinical data
☒	☐ Dual use research of concern

Methods

n/a	Involved in the study
☒	☐ ChIP-seq
☒	☐ Flow cytometry
☒	☐ MRI-based neuroimaging
